# Li-Dan-He-Ji Improves Infantile Cholestasis Hepatopathy Through Inhibiting Calcium-Sensing Receptor-Mediated Hepatocyte Apoptosis

**DOI:** 10.3389/fphar.2020.00156

**Published:** 2020-02-28

**Authors:** Huan Qin, Ling-ling Zhang, Xiao-li Xiong, Zhi-xia Jiang, Cui-ping Xiao, Lin-li Zhang, Yu-ji Wang, Yun-tao Wu, Yan-yan Qiu, Li-shan Zhou, Su-qi Yan

**Affiliations:** ^1^ Institute of Maternal and Child Health, Wuhan Children’s Hospital, Tongji Medical College, Huazhong University of Science and Technology, Wuhan, China; ^2^ State Key Laboratory of Virology, College of Life Sciences, Wuhan University, Wuhan, China; ^3^ Clinical College of Traditional Chinese Medicine, Hubei University of Chinese Medicine, Wuhan, China; ^4^ Department of Pediatrics, Wuhan NO.1 Hospital, Wuhan, China; ^5^ Department of Integrated Chinese and Western Medicine, Wuhan Children’s Hospital, Tongji Medical College, Huazhong University of Science and Technology, Wuhan, China; ^6^ Department of Social Services, Wuhan Children’s Hospital, Tongji Medical College, Huazhong University of Science and Technology, Wuhan, China; ^7^ First Clinical College of Medicine, Hubei University of Chinese Medicine, Wuhan, China; ^8^ Department of Statistics and Medical Records, Wuhan Children’s Hospital, Tongji Medical College, Huazhong University of Science and Technology, Wuhan, China

**Keywords:** infantile cholestasis, hepatocyte apoptosis, calcium-sensing receptor (CaSR), Li-Dan-He-Ji (LDHJ), forsythoside-A, emodin, chlorogenic acid

## Abstract

Infantile cholestatic hepatopathy (ICH) is a clinical syndrome characterized by the accumulation of cytotoxic bile acids in infancy, leading to serious liver cirrhosis or liver failure. The aetiology of ICH is complicated and some of them is unknown. Regardless of the aetiology, the finial pathology of ICH is hepatocyte apoptosis caused by severe and persistent cholestasis. It is already known that activation of calcium-sensing receptor (CaSR) could lead to the apoptosis of cardiomyocytes. However, the mechanism by CaSR-mediated cholestasis-related hepatocyte apoptosis is not fully understood. Li-Dan-He-Ji (LDHJ), a Traditional Chinese Medicine prescription, was developed to treat ICH. Another aim of this study was to investigate the possible mechanisms of LDHJ in cholestasis-related hepatocyte apoptosis. Using the primary hepatocytes, we first investigated the molecular mechanism of CaSR-mediated hepatocyte apoptosis in cholestasis. Then we prepared LDHJ granules and used ultra-high-performance liquid chromatography to identify the predominant drugs; confirmed the stability of the main substances; and for cell experiments screened forsythoside-A, emodin and chlorogenic acid as the three active substances of LDHJ granules. In the young rats with ANIT-induced intrahepatic cholestasis and the primary hepatocytes with TCDC-induced cholestasis-related hepatocyte apoptosis, the levels of liver injury and cholestasis-related biomarkers, calcium-sensing receptor (CaSR), hepatocyte apoptosis, Bax/Bcl-2 ratio, Cytochrome-C, caspase-3, phosphorylated-c-Jun NH_2_-terminal kinase (p-JNK)/JNK, and p-P38/P38 were all increased, while the levels of p-extracellular signal-regulated kinase (p-ERK)/ERK were decreased. However, LDHJ granules and its three active substances effectively reversed these changes. Furthermore, the three active substances reduced the increases in the intracellular calcium concentration ([Ca^2+^]_i_) and ROS levels and attenuated the dissipation of the mitochondria membrane potential in the TCDC-induced primary hepatocytes. The opposite results were obtained from the TCDC-induced primary hepatocytes treated with an agonist of CaSR (GdCl_3_) plus forsythoside-A, emodin or chlorogenic acid. Based on the results from *in vivo* and *in vitro* studies, LDHJ functions as an antagonist of CaSR to regulate hepatocyte apoptosis in cholestasis through the mitochondrial pathway and mitogen-activated protein kinase pathway.

## Introduction

Infantile cholestatic hepatopathy (ICH) refers to common and complex liver diseases characterized by cholestasis in hepatocytes and bile ducts in infancy (Morgan et al., 2013). ICH is the main cause of hospitalization for children with liver diseases and the main cause of liver transplantation for children with end-stage liver disease in China (Liu and Huang, 2015). The aetiology of ICH is complicated and includes heredity and metabolism, resulting in significant differences in the prognosis of ICH (Lee et al., 2017; Guo et al., 2018). Therefore, an early diagnosis and early treatments for ICH are particularly important. However, a timely and accurate aetiological diagnosis and the initiation of appropriate interventions are difficult to achieve in the early stage of ICH because the aetiology of ICH is unknown in approximately 5.85% to 28.21% of patients (Fischler and Lamireau, 2014; Gottesman et al., 2015). Additionally, in patients with a clear aetiology of ICH, the disease spectrum varies widely. Therefore, the clinical treatments focus on the treating symptoms to improve cholestasis. Currently, the drugs used to treat ICH are very limited, and ursodeoxycholic acid (UDCA) is a drug approved by the U.S. Food and Drug Administration (FDA) for the treatment of cholestasis. Nevertheless, the curative effects of UDCA are far from satisfactory, the complete response of some patients is not good and the clinical application of UDCA in infants is narrow due to the limited indications for the disease spectrum (Lammers et al., 2014). Despite the unclear pathogenesis of ICH, regardless of the aetiology, the outcomes of ICH are liver cirrhosis or liver failure if there are no effective treatments (Li et al., 2017). Hence, the optimal strategy for treatments targeting ICH may be to protect hepatocytes and prevent cholestasis-related hepatocyte apoptosis.

The calcium-sensing receptor (CaSR) belongs to the G protein-coupled receptor family (Tokonami et al., 2018). CaSR is involved in systemic calcium homeostasis (Rastaldi, 2011). Activated CaSR increases the intracellular calcium concentration ([Ca^2+^]_i_) and then participates in the mechanisms regulating cellular activities, including cell proliferation (Wang et al., 2018), differentiation (Singh et al., 2013) and apoptosis (Lu et al., 2013). The importance of CaSR in the pathogenesis of cardiomyocyte apoptosis has been well-documented (Paquot et al., 2017). Qi et al. reported that the activation of CaSR increases [Ca^2+^]_i_ and activates the mitochondrial pathway and mitogen-activated protein kinase (MAPK) pathway, leading to the apoptosis of cardiomyocytes (Qi et al., 2013). Actually, besides heart (Gerbino and Colella, 2018), CaSR has been detected in various tissues, such as the thyroid (Ding et al., 2013), kidney (Kwak et al., 2005), and liver (Xing et al., 2010). Therefore, CaSR may be crucial in leading to cholestasis-related hepatocyte apoptosis.

Li-Dan-He-Ji (LDHJ) was independently developed by the Department of Integrated Traditional Chinese and Western Medicine of Wuhan Children’s Hospital as a Traditional Chinese Medicine (TCM) prescription for the clinical treatment of ICH and had achieved good clinical outcomes in improving cholestasis (Yan et al., 2012; Zhang et al., 2017). However, the mechanism underlying the effects of LDHJ remains unclear. Thus, the aim of this study was to testify the effects of LDHJ on ICH and explore the possible mechanisms. We hypothesize that LDHJ regulates the expression of CaSR and subsequently regulates hepatocyte apoptosis caused by cholestasis. A commonly used agent alpha-naphthylisothiocyanate (ANIT) was used to establish the model of young rats with intrahepatic cholestasis because it induces hepatobiliary toxicity (Kossor et al., 1993). Haematoxylin and eosin (HE) staining, biochemical assays, terminal deoxynucleotidyl transferase-mediated dUTP-biotin nick end labeling (TUNEL) method, and Western blot analyses were used to investigate the role of CaSR and the potential mechanisms of the effects of LDHJ on cholestasis. Moreover, taurochenodeoxycholate (TCDC) was used to induce cholestasis-related hepatocyte apoptosis in primary hepatocytes *in vitro* (Borgognone et al., 2005). Biochemical assays, laser scanning confocal microscopy (LCSM), flow cytometry and Western Blot analyses were conducted to further explore the effects of the three active substances of LDHJ, including forsythoside-A (C_29_H_36_O_15_), emodin (C_15_H_10_O_5_), and chlorogenic acid (C_16_H_18_O_9_), and the underlying mechanisms in hepatocyte apoptosis associated with cholestasis. Our results provide preliminary scientific evidence for the rational applications of LDHJ and possible related mechanisms in the treatment of cholestasis.

## Materials and Methods

### Preparation and Testing of Experimental Drugs

LDHJ granules are composed of 11 single formula granules (CR SANJIU, Shenzhen, China), and the ratios of the single formula granule to the corresponding crude drug are also listed in **Table 1**. The equivalent rat dosage used in this study was converted according to the clinical dose for a 5kg infant. According to the technical guidelines for the quality and stability of Traditional Chinese Medicine preparations in the Chinese Pharmacopoeia, the quality testing of compound drugs is based on the predominant drug identification standards using ultral-high-performance liquid chromatography (UPLC). To identify the stability of the active ingredients in LDHJ granules, we establish the fingerprint using eight standard drugs of the predominant drugs. The predominant drugs of LDHJ were *Forsythia suspensa* (Thunb.) Vahl, *Rheum palmatum* L. (processing with rice wine) and *Artemisia capillaries* Thunb. (Zhang et al., 2019). Details of eight reference standards (National Institutes for Food and Drug Control) in the predominant drugs of LDHJ granules were shown in **Table 2**. In this study, UPLC was performed using a Waters Acquity Ultra Performance LC system (Milford, MA, USA). A WATERS water-resistant C18 (HSS T3 1.8 m×100 mm×2.1 mm) column was used to separate the components, and the mobile phase used to elute the products was a gradient of acetonitrile-1% phosphoric acid in water. The flow rate was 0.3 ml/min, the column temperature was set to 35°C, and the detection wavelength was adopted. During the full scan of wavelengths ranging from 210 to 600 nm, and the chromatograms were monitored at 277 nm. The pretreatment employed ultrasonic extraction with heated water, and the method is simple and convenient.

**Table 1:** Details of LDHJ.

Chinese materia medica		Granule forms of Individual medicinals for prescriptions			
Name	Code	Name	Code	Lot NO.	Ratio
Forsythia suspensa (Thunb.) Vahl	06171240200299005	Fructus Forsythiae	06171240200209004	1712001S	1:10
Artemisia capillaries Thunb.	06174450500799007	Herba Artemisiae Scopariae	06174450500709006	1709001S	1:10
Rheum palmatum L. (processing with rice wine)	06152310300199002	Radix Et Rhizoma Rhei (Prepared)	06152310300109612	1705001S	1:6
Schisandra sphenanthera Rehd. et Wils.	06154140200299001	Fructus Schisandrae Chinensis	06154140200209000	1707004S	1:4
Paeonia lactiflora Pall.	06153710100399005	Radix Paeoniae Rubra	06153710100309004	1711002S	1:5
Atractylodes macrocephala Koidz.	06174410500299006	Rhizoma Atractylodis Macrocephalae Praeparata	06174410500209210	1710003S	1:5
Citrus aurantium L.	06157040100299002	Fructus Aurantii Preparata	06157040100209216	1705002S	1:6
Glycyrrhiza uralensis Fisch.	06156310300299005	Radix Et Rhizoma Glycyrrhizae	06156310300209004	1712009S	1:6
Polygonum multiforum Thunb.	06152310400199001	Radix Polygoni Multiflori	06152310400109000	1709001S	1:6.7
Cinnamomum cassia Presl	06154520200199004	Ramulus Cinnamomi	06154520200109003	1710001S	1:12
Manis pentadactyia Linnaeus	06220420300199008	Manis Squama (Scalded with sand)	06220420300112229	1712003S	1:3

LDHJ, Li-Dan-He-Ji. Code of Chinese materia medica and Granule forms of individual medicinals for prescriptions is formulated by the National Standard of the People’s Republic of China: coding rules for Chinese medicines and their codes (2015). Ratio means the ratio of granule to the corresponding crude drug.

**Table 2:** Identification of the predominant drugs of LDHJ granules.

Drug granule	Standards		Content (mg/g)										Average(mg/g)	RSD (%)
	Name	Lot NO.	S1	S2	S3	S4	S5	S6	S7	S8	S9	S10		
*Herba Artemisiae Scopariae*	Chlorogenic acid	110753-201817	2.391	2.541	2.453	2.287	2.131	2.492	2.459	2.512	2.013	2.451	2.373	7.4
Fructus Forsythiae	Forsythoside-A	111810-201606	24.826	22.991	25.656	27.890	23.879	25.564	24.890	26.010	25.102	23.981	25.079	5.4
Fructus Forsythiae	Forsythin	110821-201816	5.697	5.465	5.894	5.976	5.145	5.381	5.854	5.735	6.242	5.211	5.660	6.2
Radix Et Rhizoma Rhei (prepared)	Aloe-emodin	110795-201710	0.114	0.102	0.127	0.119	0.109	0.120	0.121	0.109	0.108	0.119	0.115	6.7
Radix Et Rhizoma Rhei (prepared)	Rhein	110757-201607	0.754	0.665	0.812	0.801	0.796	0.811	0.683	0.734	0.749	0.786	0.759	6.9
Radix Et Rhizoma Rhei (prepared)	Emodin	110756-201512	0.124	0.134	0.136	0.129	0.133	0.134	0.132	0.113	0.123	0.131	0.129	5.5
Radix Et Rhizoma Rhei (prepared)	Chrysophanol	110796-201621	0.146	0.132	0.149	0.137	0.131	0.154	0.154	0.139	0.140	0.157	0.144	6.6
Radix Et Rhizoma Rhei (prepared)	Physcion	110758-201616	0.987	1.013	1.022	0.951	0.960	1.032	1.173	0.976	0.965	1.034	1.011	6.4

LDHJ, Li-Dan-He-Ji. The RSD (relative standard deviation) of eight substances in 10 batches of the predominant drugs of LDHJ granules was less than 10%.

### Animals

Four-week-old male (n=20) and female (n=20) specific pathogen-free (SPF) Sprague-Dawley (SD) rats (70–90 g) provided by the Hubei Provincial Center for Disease Control and Prevention (Certification: NO. 00138559, Wuhan, China) were used in this study. All animals were housed in the SPF level barrier system of the Laboratory Animal Center, Tongji Medical College, Huazhong University of Science and Technology (Certification: NO. 00137850, Wuhan, China) at 22°C ±1°C on an alternating 12h light/dark cycle and provided free access to food and water. All experiments were performed in accordance with the Guidelines for the Care and Use of Mammals of the National Institutes of Health and approved by the Institutional Animal Care and Use Committee at Tongji Medical College, Huazhong University of Science and Technology (2016 IACUC Number: 2313).

### Animal Modeling of ANIT-Induced Intrahepatic Cholestasis and Grouping

**Figure 1:**
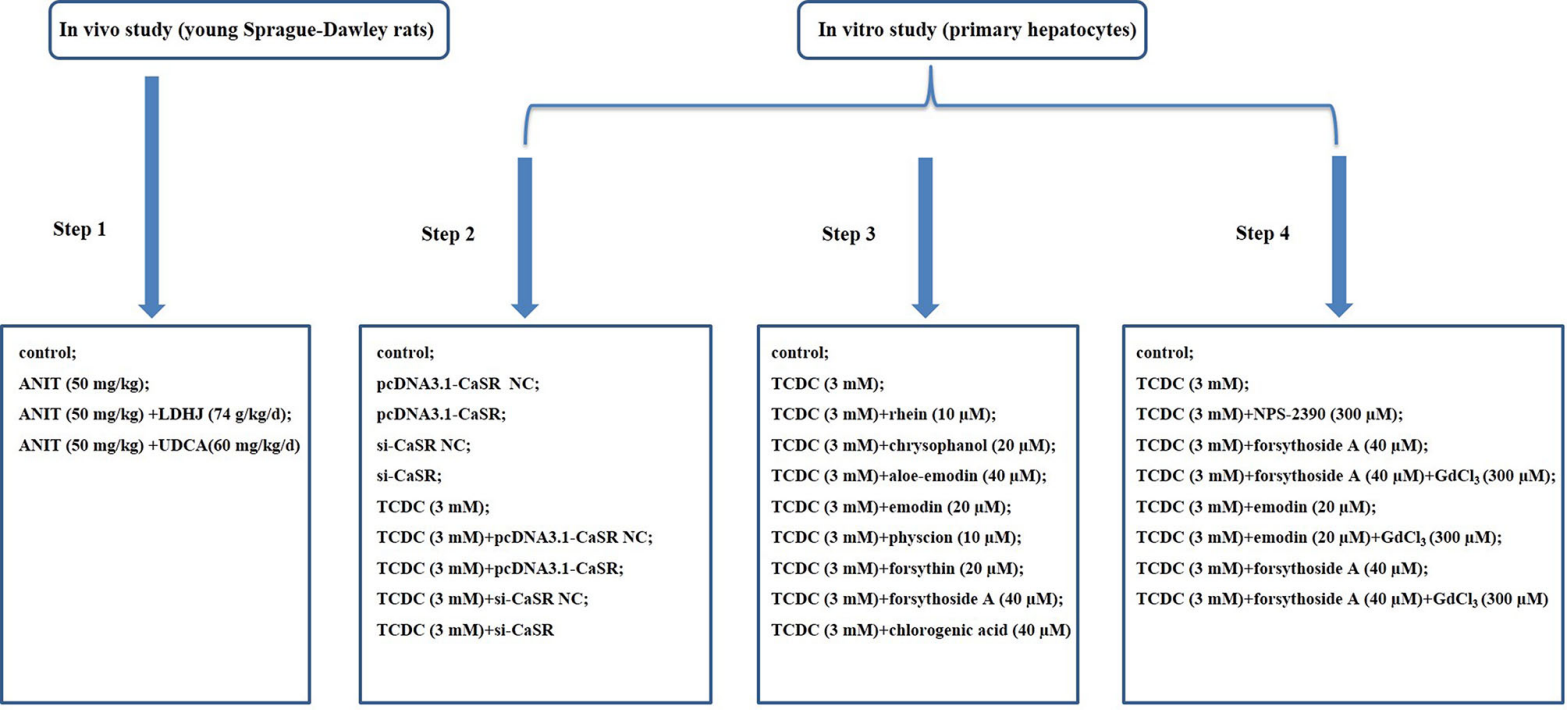
Flow chart of modeling group. Step 1: Exploration of the effects of LDHJ granules on ICH and the possible mechanism; Step 2: Verification of the key role of CaSR in cholestasis-related hepatocyte apoptosis; Step 3: Selection of the active substances of LDHJ granules for cell experiments; Step 4: Confirmation of the protective role of three active substances of LDHJ granules on the cholestasis-related hepatocyte apoptosis through regulating CaSR.

All SD rats were acclimated for seven days prior to the study and then randomly assigned into four groups with ten rats each. The ratio of males to females in each group was 1:1. Rats in the normal control group (control group) received a continuous intragastric administration of saline (0.9%) for six days. Rats in the group treated with ANIT (Sigma-Aldrich, St. Louis, MO, USA) (ANIT group) received a continuous intragastric administration of saline (0.9%) for six days and were treated with ANIT (50 mg/kg) once on the fifth day. Rats in the group treated with LDHJ granules (CR SANJIU, Shenzhen, China) (ANIT+LDHJ group) received a continuous intragastric administration of LDHJ granules (74 g/kg/d, the solvent was distilled water) for six days and were treated with ANIT (50 mg/kg) once on the fifth day. Rats in the group treated with UDCA (Meilunbio, Dalian, China) (ANIT+UDCA group) received a continuous intragastric administration of a UDCA (60 mg/kg/d) for six days and were treated with ANIT (50 mg/kg) once on the fifth day. Rats were fasted for 24 h after the last intragastric administration prior to sacrifice and then used *in vivo* experiments. The grouping information was illustrated as step 1 in **Figure 1**.

### Histopathological Analysis

Fresh liver tissues were fixed with a 4% paraformaldehyde solution for 24 h and embedded in paraffin. Paraffin-embedded tissues were cut into slices at a 4µm thickness, and then stained with HE according to the manufacturer’s instructions. Changes in liver histopathology were observed under a light microscope (Olympus, Japan). To measure the cell apoptosis rate on fresh liver tissues, terminal deoxynucleotidyl transferase-mediated dUTP-biotin nick end labeling (TUNEL) assay was performed following the manufacturer’s protocol (Roche, Switzerland) after being fixed in 4% paraformaldehyde overnight. The nucleus was stained blue, and the apoptotic cells were stained green. The stained cells were examined by a light microscope (Olympus, Japan). All pictures were taken at a magnification of 400 times. The percentage of TUNEL-positive cells in all DAPI-stained cells in each field were calculated using Image-Pro Plus 6.0 software.

### Extraction, Culture, and Cholestasis-Related Hepatocyte Apoptosis Model Establishment of Primary Hepatocytes

Primary hepatocytes were extracted from the livers of 4-week-old SD rats using a modified two-step collagenase perfusion technique (Reinehr et al., 2006). This study also was approved by the Institutional Animal Care and Use Committee at Tongji Medical College, Huazhong University of Science and Technology (2016 IACUC Number: 2313). Briefly, rats were anaesthetized with sodium pentobarbital (50 mg/kg, Sigma-Aldrich, MO, USA), followed by an injection of heparin (1,500 U/kg, APExBio, TX, USA) into the inferior vena cava and the introduction of a 14G catheter into the portal vein. The portal vein was perfused with oxygenated Hanks’ buffer (pH=7.47–7.50) without Ca^2+^ and supplemented with 3 g/L HEPES and 0.24 g/L EGTA. Subsequently, the livers were further perfused with EGTA-free oxygenated Hanks’ buffer (pH=7.47–7.50) containing MgSO_4_ (1 mM), CaCl_2_ (2.5 mM), and collagenase type IV (4300 U/L) for 5 min. Finally, the liver was removed and gently dissociated with a glass rod for 3–4 min to promote the mechanical separation of cells. Hepatocytes were purified from non-parenchymal cells by centrifugation at 50×g for 2 min and washed with oxygenated Hanks’ buffer containing 2.5 mM CaCl_2_ and 5 mM Tris three times. Isolated hepatocytes were cultured in RPMI medium containing 10% foetal bovine serum. Primary hepatocytes were treated with TCDC (Sigma-Aldrich, St. Louis, MO, USA) to establish the cholestasis-related hepatocyte apoptosis model, as described in a previous study (Borgognone et al., 2005). The optimal concentration and duration of TCDC were selected by both hepatocyte injury and cholestasis-related biochemical biomarkers, which represents the highest degree of liver injuries.

### CaSR Upregulated and Downregulated Primary Hepatocyte Models Establishment

Overexpression and RNAi experiments are widely used because of their direct and effective gene function validation. pcDNA3.1-CaSR, pcDNA3.1 (pcDNA3.1-CaSR NC), specific small interfering RNA (siRNAs) against CaSR and negative control siRNA (si-CaSR NC) were all obtained from RiboBio (Guangzhou, China). The target gene of CaSR was synthesized by DNA sequencing (CaSR accession number: XM_008842932.2) and amplified by PCR using the specific primers 5'AAAGATCCGGTACCGCTAGCGGATCCATGCTTTAATTTTCATTTGCTACC3' and 5'TCGAAGCGGCCGGCCGAGATCGAAgcttTTGTTGGAAATAATTTTTATTTAACA3' (KpnI F and XhoI R sites are underlined). The sequences of CaSR-si1, CaSR-si2 and CaSR-si3 in this study were as follows, siRNA-1 GCAACTGCTCTGAGCACAT, siRNA-2 GCGCATGCCCTACAAGATA, and siRNA-3 CCAAGATACCCACCAGCTT. CaSR overexpression cells were established by transfecting pcDNA3.1-CaSR into primary hepatocytes treated with or without TCDC. CaSR knockdown cells were established by transfecting si-CaSR into primary hepatocytes treated with or without TCDC.The lipofectamine 3000 reagent (Invitrogen, USA) was used for cell transfection. After transfection for 48 h, the harvested cells were used to the following experiments. The grouping information was illustrated as step 2 in **Figure 1**.

### Evaluation of the Eight Components of LDHJ granules for Cell Experiments

Primary hepatocytes treated with TCDC were further administrated with eight components of LDHJ granules for 24 h respectively. The less cytotoxicity (TCDC-stimulated primary hepatocyte inhibition ratio ≤ 20%) with maximum concentration of eight components were determined. The details were as follows: the TCDC+rhein group: 10 µM rhein (Sigma-Aldrich, St. Louis, MO, USA); the TCDC+chrysophanol group: 20 µM chrysophanol (Solarbio, Beijing, China); the TCDC+aloe-emodin group: 40 µM aloe-emodin (Solarbio, Beijing, China); the TCDC+emodin group: 20 µM emodin (purity ≥ 90%, lot No.043K35051V, Sigma-Aldrich, St. Louis, MO, USA); the TCDC+physcion group: 10 µM physcion (Solarbio, Beijing, China); the TCDC+forsythin group: 20 µM forsythin (Solarbio, Beijing, China); the TCDC+forsythoside-A group: 40 µM forsythoside-A (purity ≥ 98%, lot No.615A022, Solarbio, Beijing, China); the TCDC+chlorogenic acid group: 40 µM chlorogenic acid (purity ≥ 95%, lot No.WXBC4106V, Sigma-Aldrich, St. Louis, MO, USA). Primary hepatocytes treated with or without 3 mM TCDC were separately the model group (TCDC group) and the normal control group (control group). The grouping information was illustrated as step 3 in **Figure 1**. According to the effects of eight components of LDHJ granules on CaSR expression and cell apoptosis, forsythoside A, emodin and chlorogenic acid were selected and used to the following analyses.

### Cell Treatments

Cells were randomly distributed into nine groups: the control group: cells were cultured using standard procedures; the TCDC group: cells were treated with 3 mM TCDC for 3 h; the TCDC+NPS-2390 group: cells were treated with 3 mM TCDC for 3 h, followed by the administration of 300 µM NPS-2390 (antagonist of CaSR, R&D Systems, MN, USA) for 1 h; the TCDC+forsythoside-A group: cells were treated with 3 mM TCDC for 3 h, followed by the administration of 40 µM forsythoside-A for 24 h; the TCDC+forsythoside-A+GdCl_3_ group: cells were treated with 3 mM TCDC for 3 h, followed by the administration of 40 µM forsythoside-A for 24 h plus 300 µM GdCl_3_ (agonist of CaSR, Sigma-Aldrich, St. Louis, MO, USA) for 1 h; the TCDC+emodin group: cells were treated with 3 mM TCDC for 3 h, followed by the administration of 20 µM emodin for 24 h; the TCDC+emodin+GdCl_3_ group: cells were treated with 3 mM TCDC for 3 h, followed by the administration of 20 µM emodin for 24 h plus 300 µM GdCl_3_ for 1 h; the TCDC+chlorogenic acid group: cells were treated with 3 mM TCDC for 3 h, followed by the administration of 40 µM chlorogenic acid for 24 h; the TCDC+chlorogenic acid+GdCl_3_ group: cells were treated with 3 mM TCDC for 3 h, followed by the administration of 40 µM chlorogenic acid for 24 h plus 300 µM GdCl_3_ for 1 h. The grouping information was illustrated as step 4 in **Figure 1**.

### Cell Counting Kit-8 Assay

Cell viability was determined using a Cell Counting Kit-8 (CCK-8) (Beyotime, China) assay according to the manufacturer’s protocols. Cells were plated in 96-well plates (1×10^4^ cells per well) and cultured in an incubator with a 5% CO_2_ atmosphere at 37°C for 24 h. After treatment with the designated drugs, cells in each well were incubated with 10 µl of CCK-8 reagent for another 2 h. The absorbance (λ/nm=450) of each well was assessed using a microplate reader (Diatek, Wuxi, China) to determine cell viability.

### Flow Cytometry Assay

The cells were digested using 0.25% trypsin, and then washed with ice-cold PBS buffer. Subsequently, the cells were incubated in Annexin V-FITC and propidium iodide (PI) for 30 min at room temperature. Cells were measured within 1 h after dyeing based on Annexin V/PI double dye kit (Sungene Biotech, Tianjin, China). Cell apoptosis was assessed by flow cytometry.

### Measurement of [Ca^2+^]_i_ Using LSCM in Primary Hepatocytes

Primary hepatocytes were seeded in 6-well plates and cultured for 24 h. After treatment with the indicated drugs for 24 h, primary hepatocytes were stained with 5 mM Rhod-2 AM and suspended in PBS. LSCM (Zeiss, Oberkochen, Germany) was employed to monitor the fluorescence intensity (excitation wavelength of 549 nm and emission wavelength of 578 nm).

### Measurement of ROS Production in Primary Hepatocytes

ROS levels in the cells were measured using a Reactive Oxygen Species Assay Kit (Beyotime, Shanghai, China) according to the manufacturer’s instructions. Briefly, primary hepatocytes were inoculated in 6-well plates. After treatment with the indicated drugs, cells were suspended in serum-free culture medium supplemented with 10 µM DCFH-DA and incubated at 37°C for 20 min in the dark. Finally, cells were rinsed with serum-free culture medium. ROS generation was measured using a flow cytometer at the 488/522 nm.

### Measurement of the MMP in Primary Hepatocytes

A mitochondrial membrane potential assay kit with JC-1 (Beyotime, Shanghai, China) was used to measure the changes in the MMP (Δψm, JC-1 polymer/monomer ﬂuorescence ratio) in primary hepatocytes according to the manufacturer’s protocols. In living cells, JC-1 aggregates in the matrix to form polymers and presents red fluorescence when the MMP is high, while in apoptotic cells, JC-1 is presented as a monomer and exhibits green fluorescence when the MMP is low. After treatment with the designated drugs, the JC-1 working solution was added to the culture medium of cells growing on slides and incubated at 37°C for 20 min in the dark. Subsequently, cells were rinsed with PBS three times. The changes of MMP were assessed using a flow cytometer (excitation wavelength of 488 nm and emission wavelength of 525 nm ±20 nm/585 nm ±20 nm) and analyzed by Flow Jo software.

### Analysis of Biochemical Parameters

Following anaesthetization, serum samples were collected from the eyes of each rat. The levels of alanine aminotransferase (ALT), aspartate aminotransferase (AST), alkaline phosphatase (ALP), γ-glutamyl transpeptidase (γ-GT), total bilirubin (TBIL), direct bilirubin (DBIL) , and total bile acid (TBA) were measured using the corresponding commercially available kits (Nanjing Jiancheng Bioengineering Institute, China) according to the manufacturer’s protocols, and then analyzed with a microplate reader. The levels of these biomarkers were also detected in the supernatants of primary hepatocytes.

### Western Blot Analysis

RIPA buffer containing the complete protease inhibitor was applied to lyse the tissues and cells. Lysates were centrifuged at 12,000 rpm for 5 min at 4°C. The concentrations of proteins in the supernatants were quantified using a BCA protein assay kit (Aspen, Canada, USA). Protein samples were resolved on 10% SDS-PAGE gels and transferred to PVDF membranes. Then, the membranes were blocked with 5% nonfat dried milk for 1 h, followed by successive incubations with the primary antibodies (**Table 3**) and the corresponding horseradish peroxidase (HRP)-conjugated secondary antibodies. GAPDH served as a loading control. Protein bands were visualized using enhanced chemiluminescence (ECL) reagents and analyzed using an imaging system (QImaging, Surrey, Canada).

**Table 3:** The primary antibodies used for western blot analysis.

Antibody	Species	Manufacturer	Dilution ratio
GAPDH	Rabbit	Abcam	1:10,000
CaSR	Mouse	Abcam	1:500
Bax	Rabbit	Cell Signaling Technology	1:2,000
Bcl-2	Rabbit	Abcam	1:1,000
Cytochrome-C	Rabbit	Abcam	1:1,000
Cleaved caspase-3	Rabbit	Abcam	1:500
ERK	Rabbit	Cell Signaling Technology	1:2,000
p-ERK	Rabbit	Cell Signaling Technology	1:1,000
P38	Rabbit	Abcam	1:2,000
p-P38	Rabbit	Cell Signaling Technology	1:1,000
JNK	Rabbit	Proteintech	1:2,000
p-JNK	Rabbit	Abcam	1:1,000

CaSR, calcium-sensing receptor; ERK, Extracellular signal-regulated kinase; JNK, c-Jun NH_2_-terminal kinase; p-, phosphorylated-.

### Statistical Analysis

Each experiment was conducted three times. All data were analyzed using GraphPad Prism 7.00 software (GraphPad Software, Inc.) and presented as the means ± standard deviations (SD). Significant differences between multiple groups were analyzed using one-way ANOVA. *P* values less than 0.05 were considered statistically significant.

## Results

### Identification of LDHJ Granules

**Figure 2:**
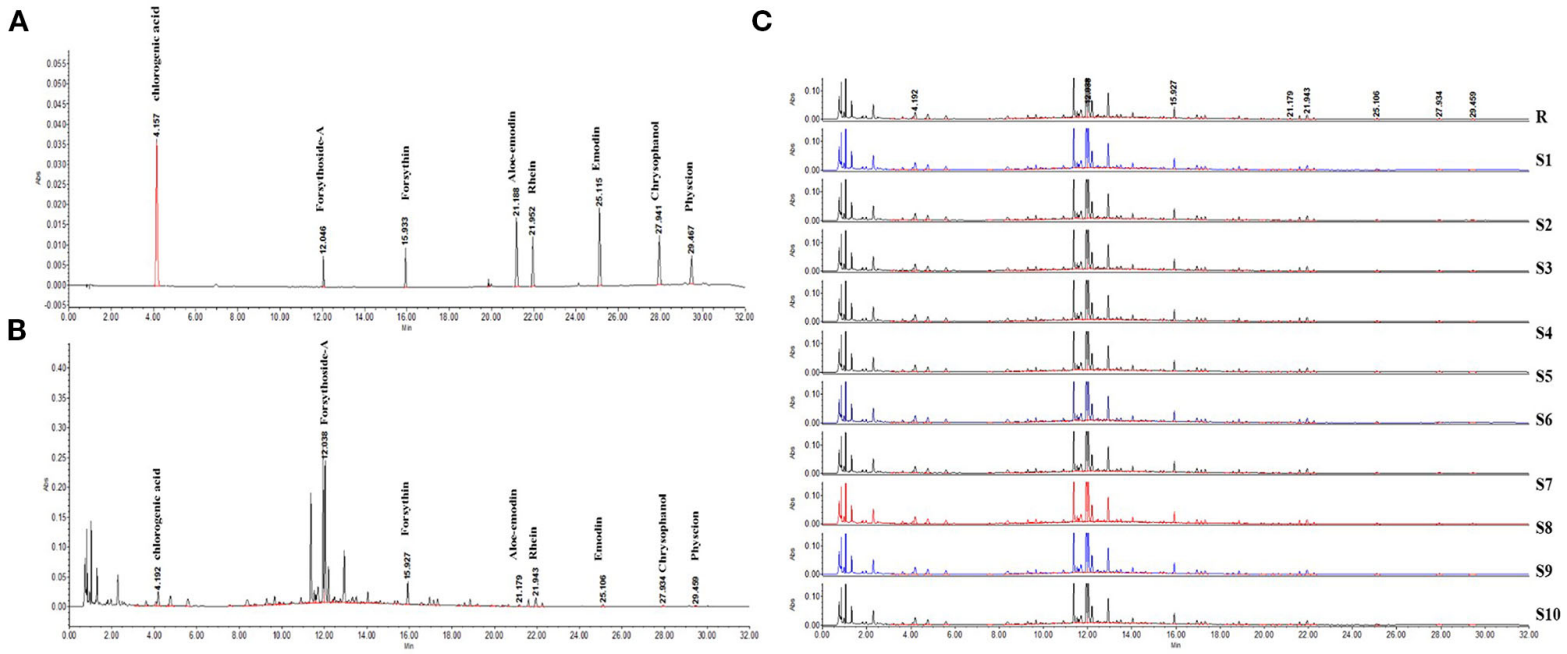
UPLC chromatogram of the predominant drugs of LDHJ granules. **(A)** UPLC fingerprint of the predominant drugs of LDHJ granules. **(B)** Chromatograms of a mixed standard solution. **(C)** UPLC chromatogram of 10 different batches of the predominant drugs of LDHJ granules.

In this study, we successfully established a standard fingerprint for the identification of the predominant drugs. Results confirmed that the contents of eight main substances (**Table 2**) in predominant drug of LDHJ granules were stable and reached the requirements of Chinese Pharmacopoeia (**Figures 2A–C**), and therefore LDHJ granules as qualified samples can be used in *in vivo* study.

### LDHJ Granules Improved the ANIT-Induced Intrahepatic Cholestasis, Reduced CaSR Expression, and Inhibited Hepatocyte Apoptosis in the Young Rats

#### Plasma Biomarkers Associated With Cholestasis and Liver Injuries

The levels of various biomarkers were detected to further confirm the protective role of LDHJ granules in ANIT-induced hepatic injuries. As displayed in **Table 4**, rats treated with ANIT presented significantly increased levels of both cholestasis and liver injuries related-biomarkers, including ALT, AST, γ-GT, ALP, TBIL, DBIL, and TBA, compared with the normal control group (*P* < 0.01). However, the increased levels of these markers were attenuated by LDHJ granules and UDCA (*P* < 0.05, *P* < 0.01). Thus, LDHJ granules ameliorated ANIT-induced intrahepatic cholestasis and liver injuries.

**Table 4:** Effects of LDHJ granules on plasma biomarkers associated with cholestasis and liver injuries in the young rats.

Group	ALT	AST	γ-GT	ALP	TBIL	DBIL	TBA
control	19.79 ± 2.83	30.42 ± 4.28^**^	13.75 ± 1.66^##^	30.14 ± 1.63^#^	44.95 ± 2.78^#^	19.71 ± 1.49^#^	47.73 ± 4.70^#^
ANIT	81.55 ± 7.62	101.03 ± 4.63^**^	60.52 ± 3.85^##^	63.28 ± 3.51^#^	110.06 ± 7.25^#^	51.35 ± 4.16^#^	228.16 ± 12.29^#^
ANIT+LDHJ	22.95 ± 3.32	36.25 ± 2.26^**^	19.01 ± 2.60^##^	34.05 ± 1.82^#^	48.59 ± 2.65^#^	24.04 ± 1.28^#^	77.38 ± 5.29^#^
ANIT+UDCA	54.36 ± 11.48	76.70 ± 8.03^**^	49.48 ± 6.71^##^	49.96 ± 7.96^#^	82.4 ± 10.71^#^	37.65 ± 4.85^#^	151.4 ± 9.59^#^

Data are presented as the means±SD. n=10. LDHJ, Li-Dan-He-Ji; ALT, alanine aminotransferase; AST, aspartate aminotransferase; γ-GT, γ-glutamyl transpeptidase; ALP, alkaline phosphatase; TBIL, total bilirubin; DBIL, direct bilirubin; TBA, total bile acid. **P < 0.01 vs the control group; ^#^P < 0.05 and ^##^P < 0.01 vs the ANIT group.

#### Histological Analysis

**Figure 3:**
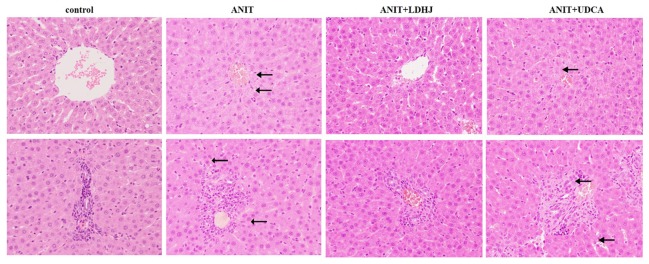
Morphologic photos of rat's hepatic tissue. H&E staining. Original magnification: ×400. There is bile deposition in the area indicated by the black arrow.

HE staining was conducted to investigate the effects of LDHJ granules on the ANIT-induced hepatic injuries. As shown in **Figure 3**, the tissue structure of the central vein and portal area of the normal liver was intact in the normal control group. In the central vein and portal area of the ANIT-treated group, there was hepatocyte necrosis around the central vein, inflammatory cell infiltration could be seen in the portal area. Compared with the ANIT-treated group, the central vein and portal area in ANIT+LDHJ group were significantly improved, the necrotic cells around the central vein decreased and the inflammatory cells in the portal area decreased. The central vein and portal area in ANIT+UDCA group showed that there was still hepatocyte necrosis around the central tubule, inflammatory cell infiltration and bile duct hyperplasia in the portal area, and vacuolar degeneration in hepatocyte. However, LDHJ granules attenuated ANIT-induced cholestasis and promoted the hyperplasia of bile duct, indicating the protective effects of LDHJ granules on the ANIT-induced hepatic injuries.

**Figure 4:**
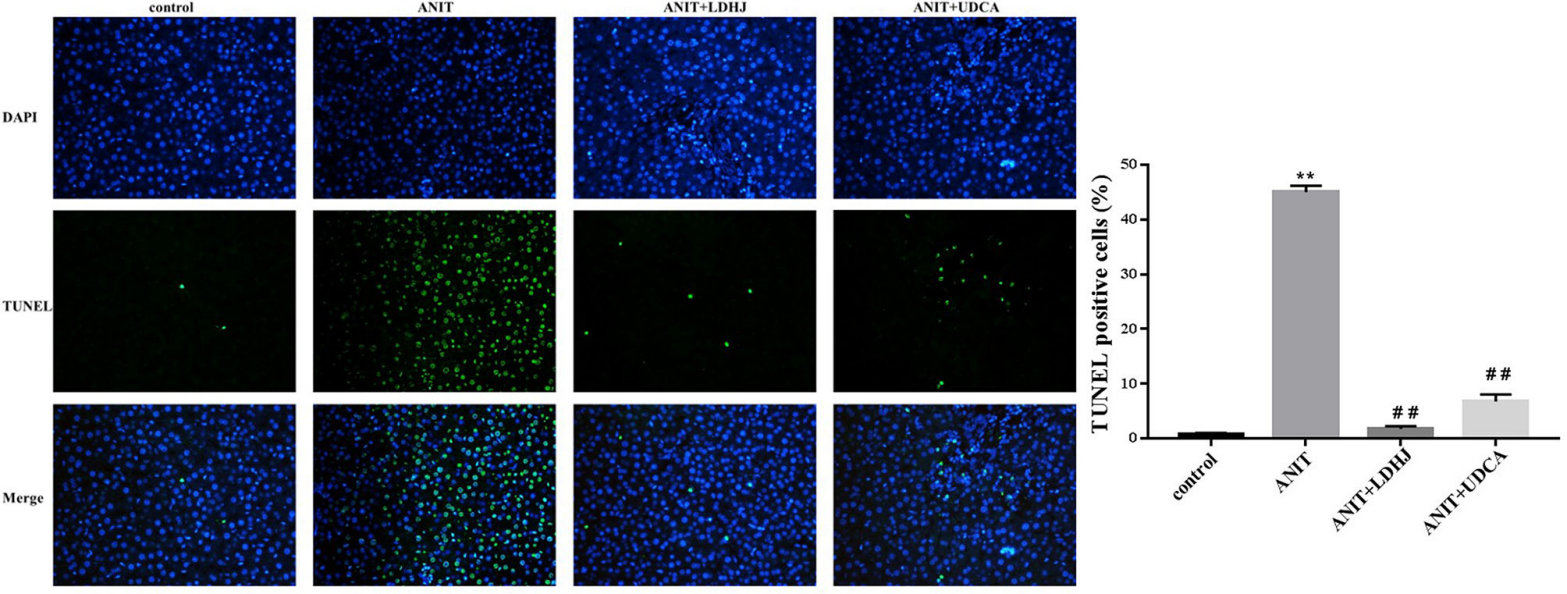
TUNEL staining in the liver from the rats. Original magnification: ×400. The nucleus was stained blue, and the apoptotic cells were stained green. Five random fields per section were examined in each experiment. **P < 0.01 vs the control group; ^##^ *P* < 0.01 vs the ANIT group.

TUNEL staining was conducted to estimate the hepatocyte apoptosis in rats. As shown in **Figure 4**, the apoptosis cells were stained green. The apoptotic rate in the ANIT group was significantly elevated compared with the normal control group (*P* < 0.01). However, the apoptotic rate was markedly reduced in the ANIT+LDHJ group compared with the ANIT group (*P* < 0.01).

#### Hepatic CaSR Expression

**Figure 5:**
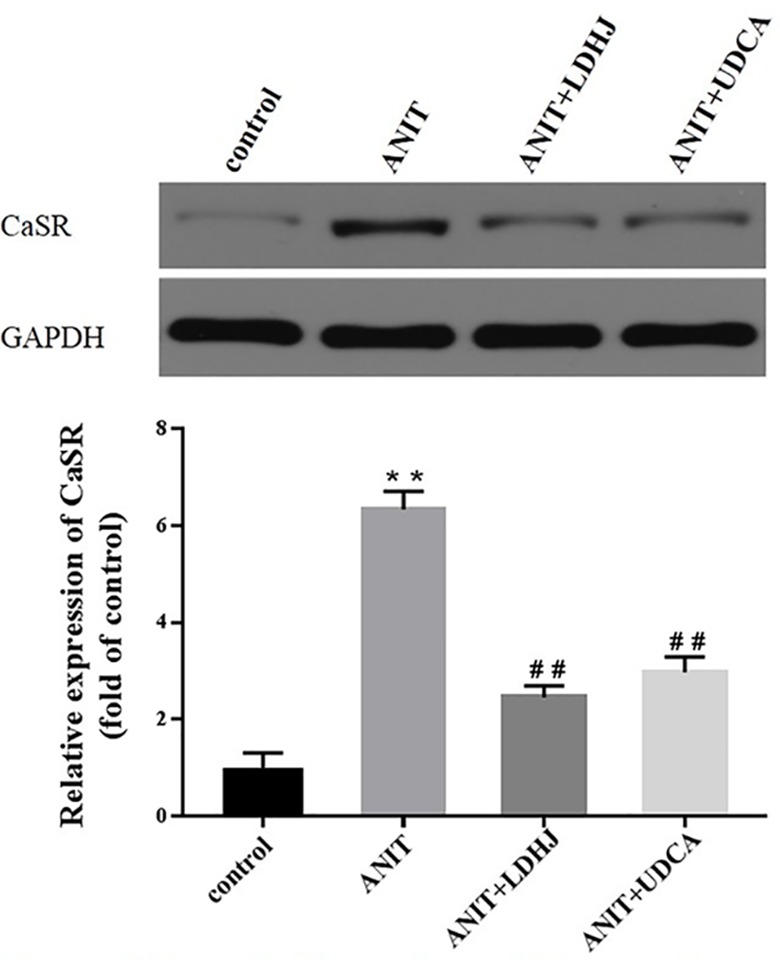
Effects of LDHJ granules on CaSR expression *in vivo*. The levels of CaSR were detected in the young rats using the western blot analysis. GAPDH served as the internal control. **P < 0.01 vs the control group; ^##^P < 0.01 vs the ANIT group.

Western blot analyses were performed to assess the effects of LDHJ granules on CaSR levels in the young rats with cholestasis. As shown in **Figure 5**, the levels of CaSR protein were markedly increased in the ANIT-treated group compared with the normal control group (*P* < 0.01). Additionally, compared with the ANIT-treated group, LDHJ granules, and UDCA significantly decreased CaSR expression (*P <*0.01). Based on these results, LDHJ granules inhibited the expression of CaSR in the young rats with ANIT-induced cholestasis.

#### Hepatic Levels of Apoptosis-Related Proteins Involved in the Mitochondrial Pathway

**Figure 6:**
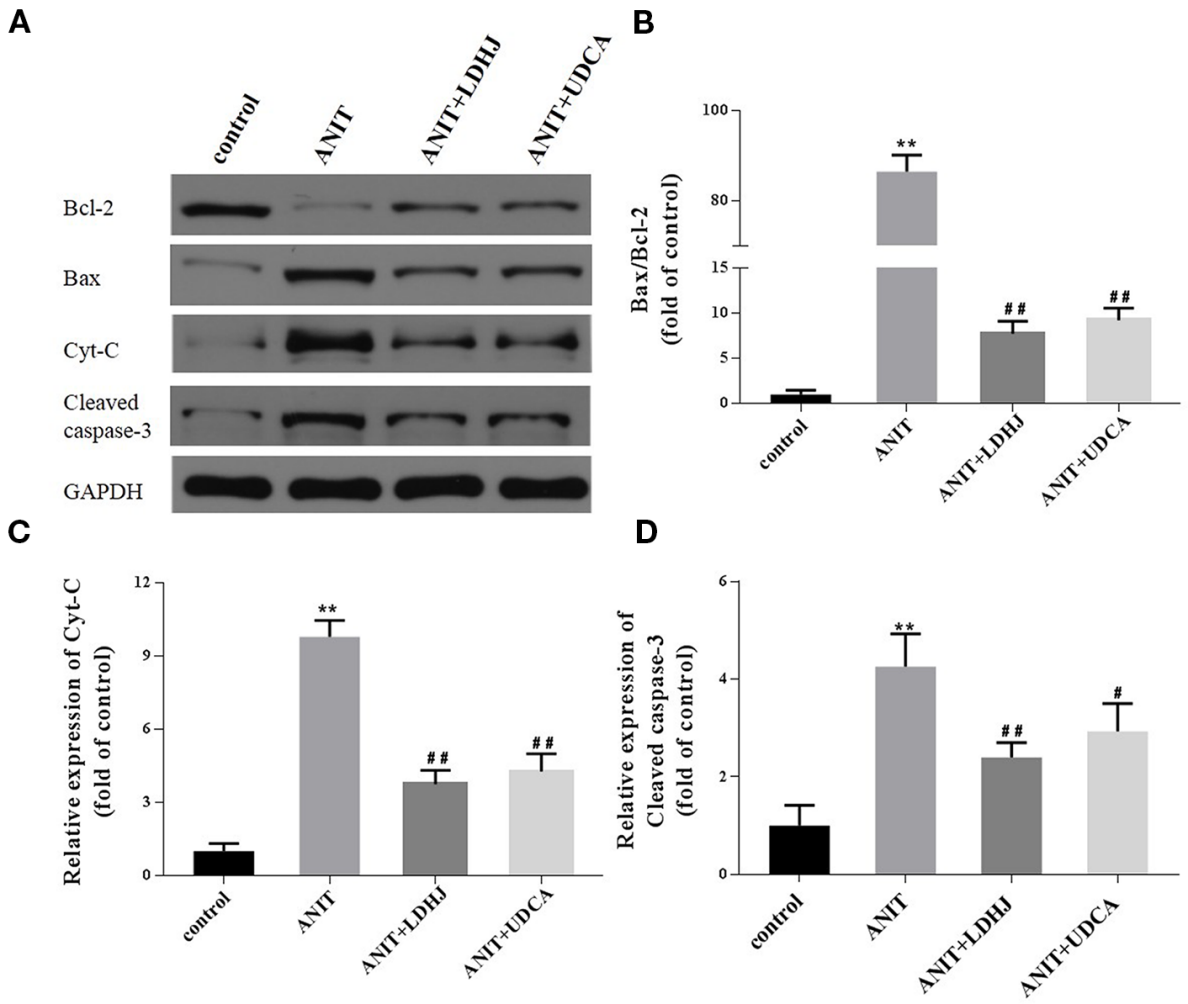
Effects of LDHJ granules on apoptosis-related proteins involved in the mitochondrial pathway *in vivo*. **(A–D)** The levels of Bax, Bcl-2, Cyt-C, and cleaved caspase-3 in the young rats were analyzed using western blot analysis. GAPDH served as the internal control. **P < 0.01 vs the control group; ^#^ *P* < 0.05, ^##^ *P* < 0.01 vs the ANIT group.

Western blot analyses were conducted to assess the effects of LDHJ granules on cell apoptosis in the intrahepatic cholestasis models. The ratio of Bax/Bcl-2 and the levels of Cyt-C and cleaved caspase-3 were remarkably increased in the ANIT-treated group compared with the normal control group (**Figures 6A–D**, *P* < 0.01). However, LDHJ granules and UDCA inhibited the increases in the ratio of Bax/Bcl-2 and the levels of the Cyt-C and cleaved caspase-3 proteins (**Figures 6A–D**, *P* < 0.05, *P* < 0.01). Collectively, LDHJ granules suppressed the expressions of apoptosis-related proteins involved in the mitochondrial pathway in the young rats with ANIT-induced cholestasis.

#### Hepatic Levels of Apoptosis-Related Proteins Involved in the MAPK Pathway

**Figure 7:**
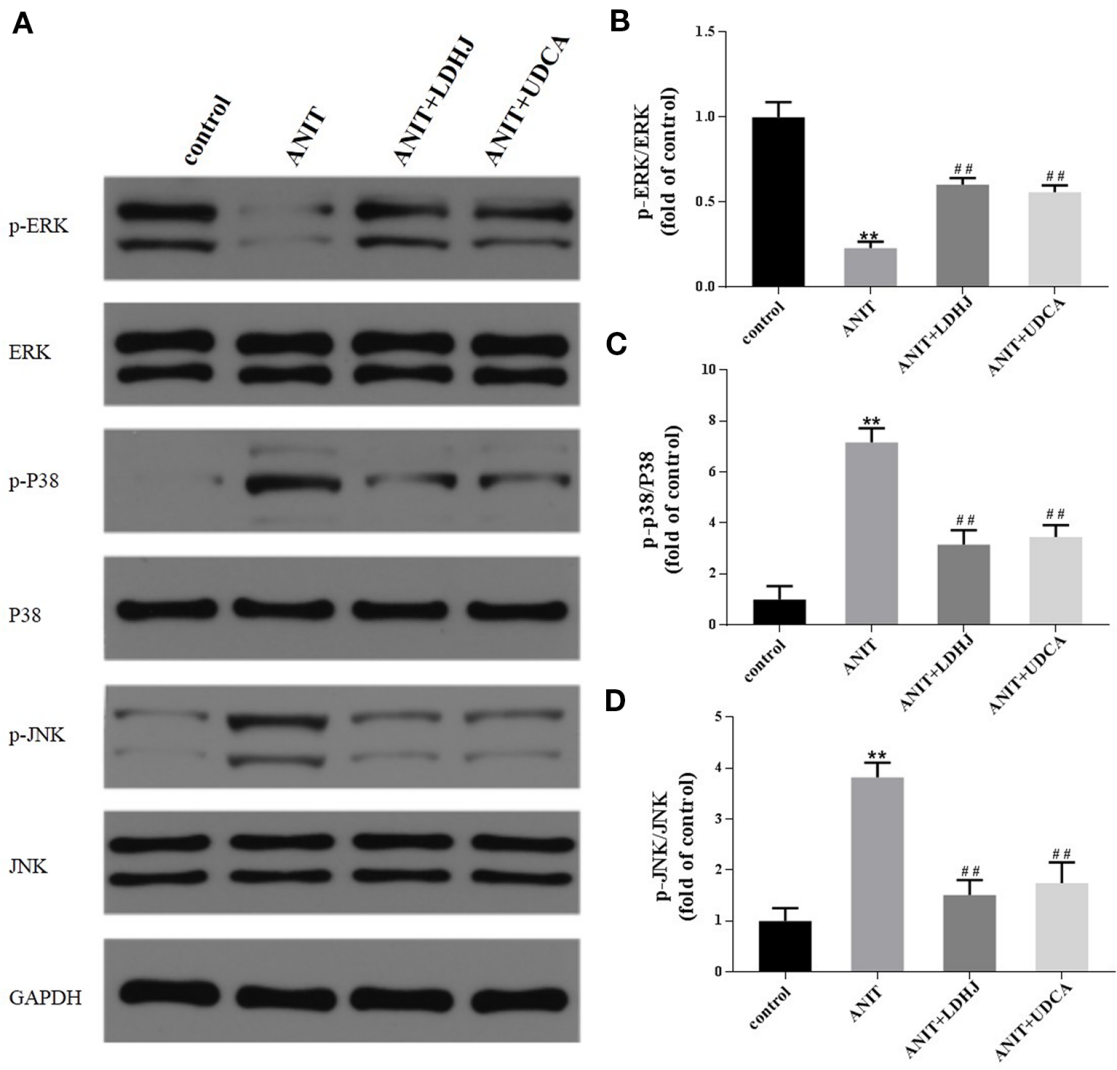
Effects of LDHJ granules on apoptosis-related proteins involved in the MAPK pathway *in vivo*. **(A–D)** The levels of p-ERK, ERK, P-P38, P38, p-JNK, and JNK in the young rats were analyzed using western blot analysis. GAPDH served as the internal control. **P < 0.01 vs the control group; ^##^P < 0.01 vs the ANIT group.

The levels of MAPK proteins were analyzed using Western blot analyses to further investigate the potential mechanisms by which LDHJ granules exerts its inhibitory effect on hepatocyte apoptosis in cholestasis. Compared with the normal control group, the levels of p-ERK/ERK were significantly decreased in the ANIT-treated group, while the levels of p-JNK/JNK and p-P38/P38 were significantly increased (**Figures 7A–D**, *P* < 0.01). However, the opposite results were obtained in the LDHJ granules- and UDCA-treated groups (**Figures 7A–D**, *P* < 0.01). Taken together, LDHJ granules inhibited the activation of JNK and P38, but stimulated the activation of ERK.

### Verification of the Key Role of CaSR in Cholestasis-Related Hepatocyte Apoptosis

#### Screening the Optimal Concentration and Duration of TCDC for the Cholestasis-Related Hepatocyte Apoptosis Model Establishment

**Figure 8:**
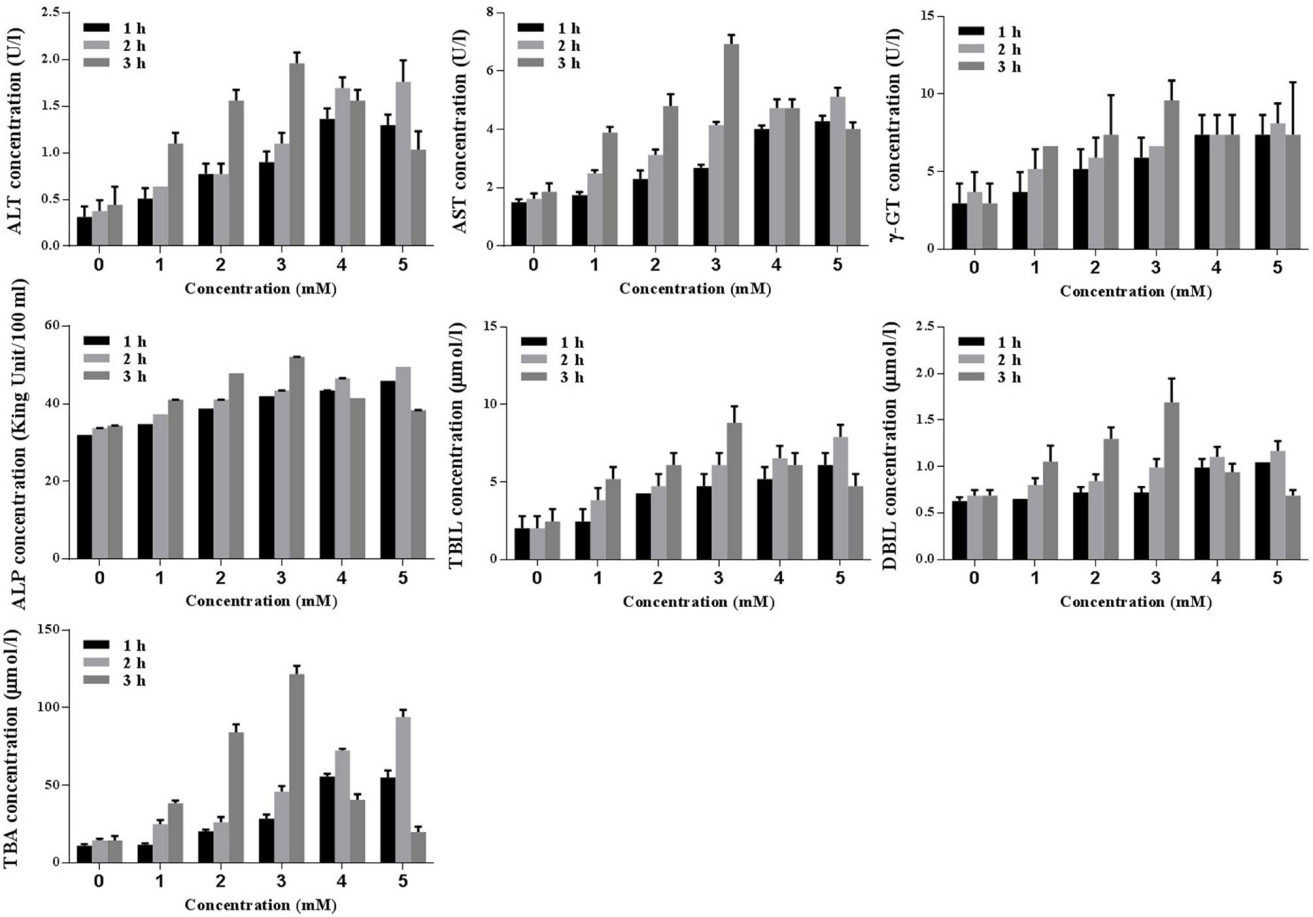
Screening the optimal concentration and duration of TCDC in primary hepatocytes for the cholestasis-related hepatocyte apoptosis model establishment. ALT, alanine aminotransferase; AST, aspartate aminotransferase, γ-GT, γ-glutamyl transpeptidase; ALP, alkaline phosphatase; TBIL, total bilirubin; DBIL, direct bilirubin; TBA, total bile acid.

Biochemical analyses (ALT, AST, γ-GT, ALP, TBIL, DBIL, and TBA) were conducted to identify the optimal concentration and duration of treatments with TCDC. Primary hepatocytes were treated with various concentration of TCDC (0, 1, 2, 3, 4, and 5 mM) for different times (1, 2, and 3 h). As revealed in **Figure 8**, a treatment with 3 mM TCDC for 3 h was the optimal cell experimental condition because the levels of biomarkers showed the most obvious cholestasis and hepatic injuries in the primary hepatocytes, accompanied with hepatocyte apoptosis (**Figure 10**).

#### The Association Between the Changes of Hepatocyte CaSR Expression and the Progression of Hepatocyte Apoptosis

**Figure 9:**
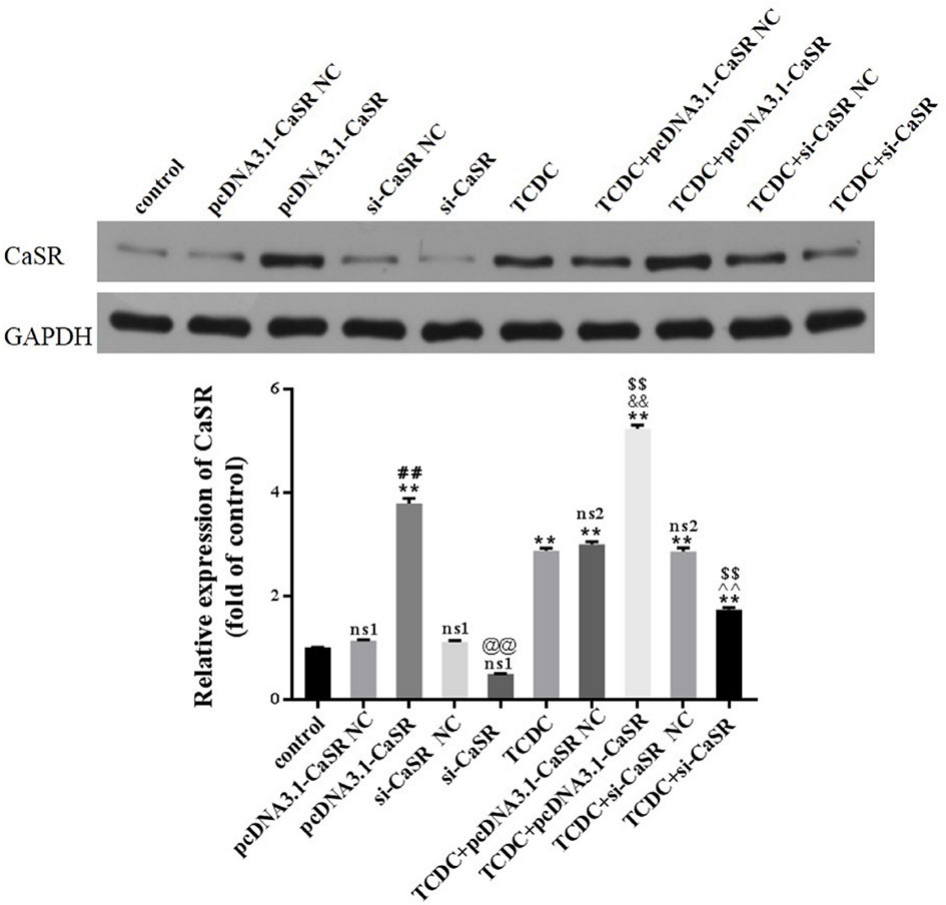
The CaSR expression in different primary hepatocyte models. The levels of CaSR were detected in primary hepatocytes using the western blot analysis. GAPDH served as the internal control. ns1, no significant difference vs the control group; ns2, no significant difference vs the TCDC group; **P < 0.01 vs the control group; ^$$^ *P* < 0.01 vs the TCDC group; ^##^ *P* < 0.01 vs the pcDNA3.1-CaSR NC group; ^@@^ *P* < 0.01 vs the si-CaSR NC group; ^&&^ *P* < 0.01 vs the TCDC+ pcDNA3.1-CaSR NC group; ^^^^ *P* < 0.01 vs the TCDC+ si-CaSR NC group.

Western blot analysis was performed to assess the expression of CaSR levels in primary hepatocytes, as revealed in **Figure 9**. The levels of CaSR protein were markedly increased in pcDNA3.1-CaSR group (vs. pcDNA3.1-CaSR NC group, *P* < 0.01) and TCDC+pcDNA3.1-CaSR group (vs. TCDC+pcDNA3.1-CaSR NC group, *P* < 0.01). Additionally, si-CaSR group (vs. si-CaSR NC group, *P* < 0.01) and TCDC+si-CaSR group (vs. TCDC+si-CaSR NC group, *P* < 0.01) were significantly decreased CaSR expression. It is successfully constructed that the CaSR over- and down-expressed in primary hepatocytes treated with or without 3 mM TCDC.

Compared with normal control group, no differences in the two NC groups without TCDC-treated and si-CaSR group (*P* ≥ 0.05). Compared with TCDC group, no differences in the two NC groups with TCDC-treated (*P* ≥ 0.05). Compared with the normal control group, the CaSR level was markedly increased in pcDNA3.1-CaSR group and all five groups treated with TCDC (*P* < 0.01). Compared with the TCDC-treated group the CaSR protein was significantly decreased in TCDC+si-CaSR group and was significantly increased in TCDC+pcDNA3.1-CaSR group (*P* < 0.01).

**Figure 10:**
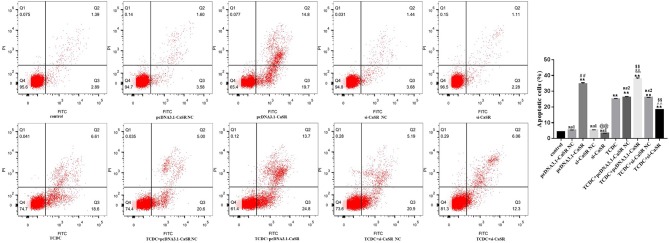
Apoptosis in different primary hepatocyte model. Annexin V/PI double staining kit was performed. Cell apoptosis were detected by flow cytometry. ns1, no significant difference vs the control group; ns2, no significant difference vs the TCDC group; **P < 0.01 vs the control group; ^$$^ *P* < 0.01 vs the TCDC group; ^##^ *P* < 0.01 vs the pcDNA3.1-CaSR NC group; ^@@^ *P* < 0.01 vs the si-CaSR NC group; ^&&^ *P* < 0.01 vs the TDCD+ pcDNA3.1-CaSR NC group; ^^^^ *P* < 0.01 vs the TCDC+ si-CaSR NC group.

Hepatocyte apoptosis were estimated by flow cytometry, as revealed in **Figure 10**. Compared with the normal control group, the apoptotic rate was markedly increased in pcDNA3.1-CaSR group and all five groups treated with TCDC (*P* < 0.01). The apoptotic rate was markedly decreased in TCDC+si-CaSR group and was markedly increased in TCDC+pcDNA3.1-CaSR group, compared with the TCDC-treated group (*P* < 0.01).

### Selection of the Active Substances of LDHJ Granules for Cell Experiments

**Figure 11:**
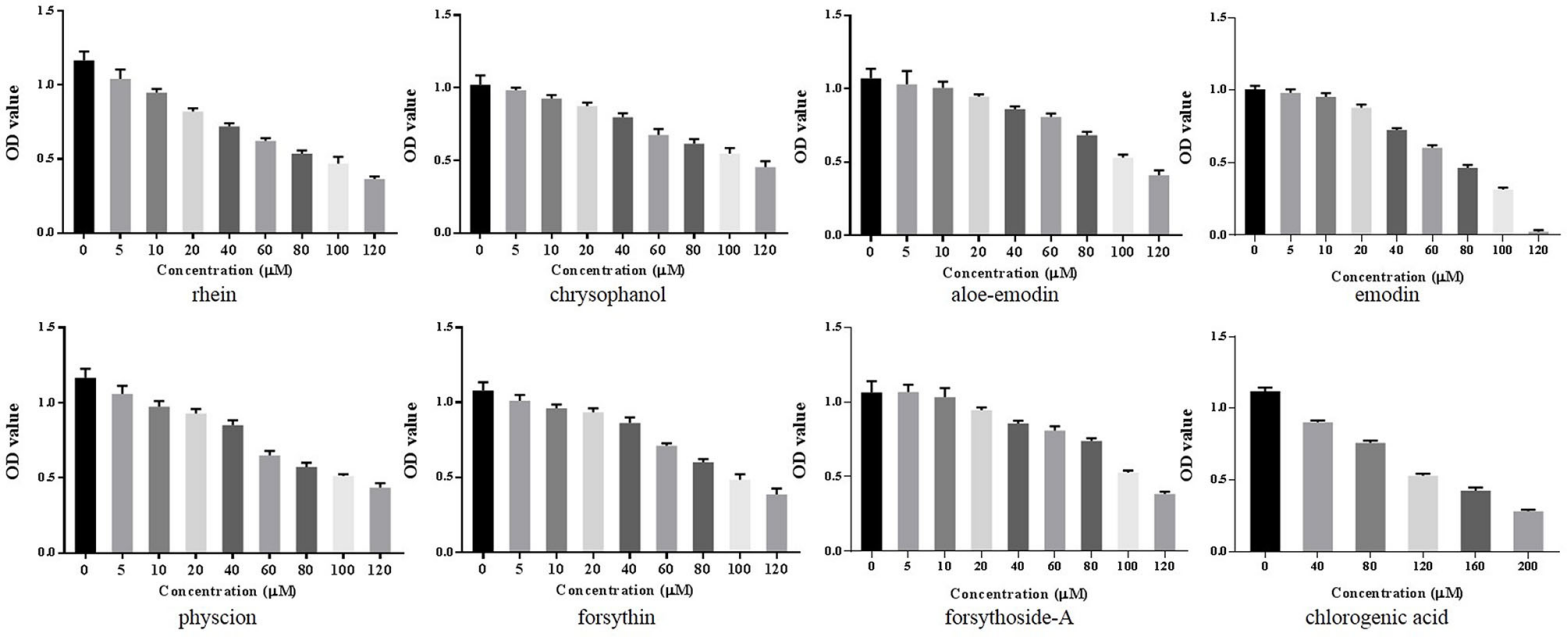
Screening the optimal concentrations of the eight main substances of LDHJ granules in TCDC-treated primary hepatocytes. Cell viability of TCDC-treated primary hepatocytes were detected using CCK-8 assay.

**Figure 12:**
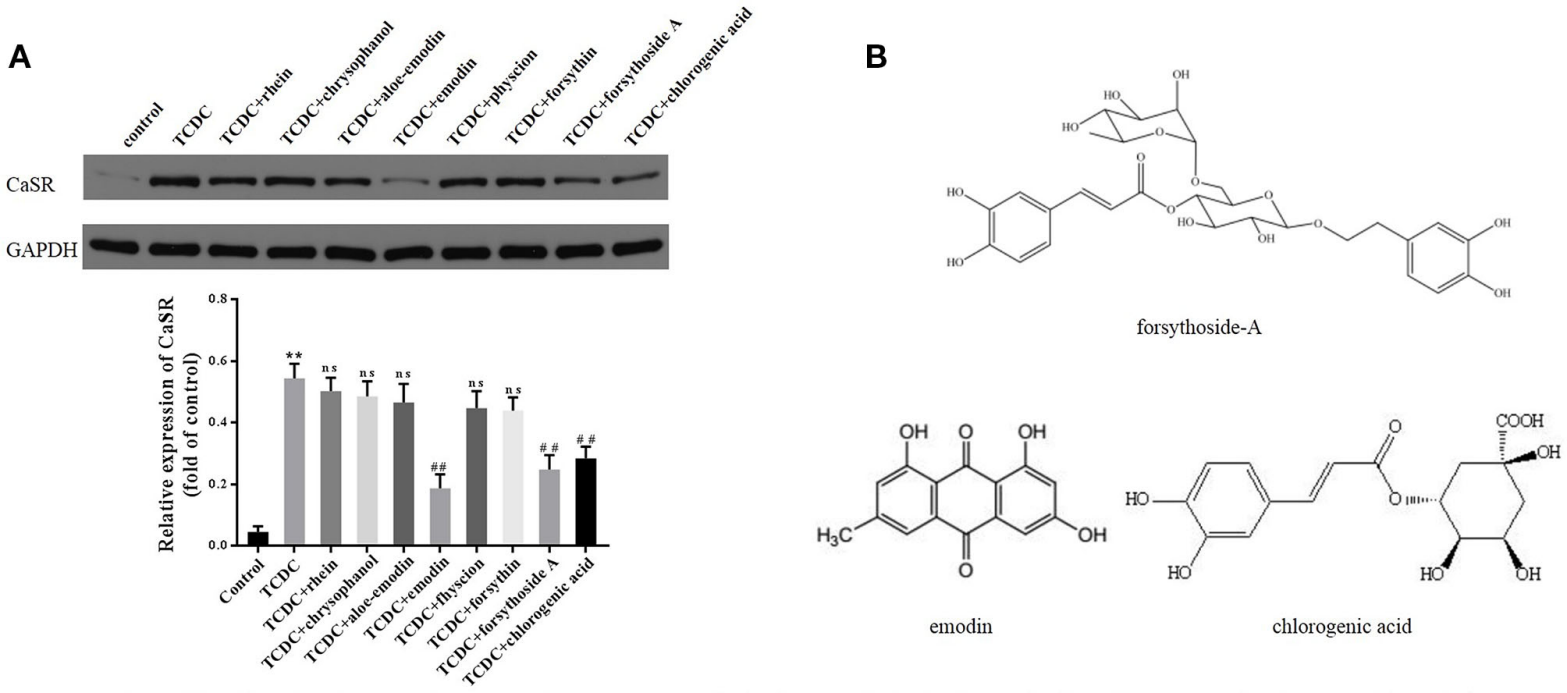
Effects of the eight main substances of LDHJ granules on CaSR expression *in vitro*. **(A)** The levels of CaSR in primary hepatocytes using western blot analysis. GAPDH served as the internal control. **P < 0.01; vs the control group; ^##^ *P* < 0.01; vs the TCDC group. ns, no significant difference vs the TCDC group. **(B)** The structure formulas of forsythoside-A, emodin, and chlorogenic acid.

**Figure 13:**
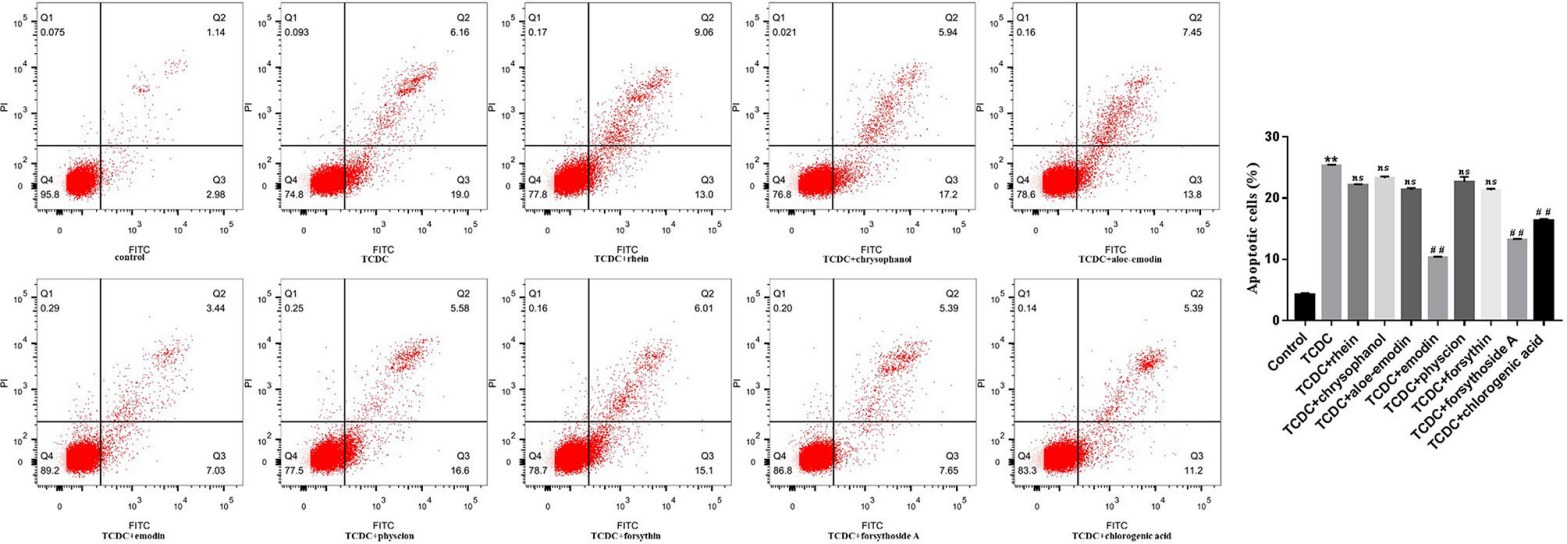
Effects of eight main substances of LDHJ granules on apoptosis *in vitro*. Annexin V/PI double staining kit was performed. Cell apoptosis were detected by flow cytometry. **P < 0.01; vs the control group; ^##^ *P* < 0.01 vs the TCDC group; ns, no significant difference vs the TCDC group.

A CCK-8 assay was conducted to identify the optimal concentrations of intervention with eight main substances of LDHJ granules. As revealed in **Figure 11**, IC_50_ value for rhein, chrysophanol, aloe-emodin, emodin, physcion, forsythin, forsythoside-A, and chlorogenic acid were separately 61.652 µM, 113.074 µM, 108.267 µM, 67.698 µM, 79.705 µM, 92.821 µM, 104.837 µM, and 112.697 µM. The maximum concentration with inhibition ratio ≤ 20% of the eight components were determined and the effects of them on CaSR expression and hepatocyte apoptosis were estimated in the next selection experiments. Compared with the TCDC group, the hepatocytic CaSR level significantly decreased and hepatocyte apoptosis remarkably improved in forsythoside-A, emodin and chlorogenic acid (**Figures 12A** and **13**, *P* < 0.01) intervention groups. However, no differences in the CaSR expression and hepatocyte apoptosis process were identified in other five components intervention groups (*P* ≥ 0.05). Thus, forsythoside A, emodin and chlorogenic acid were screened as the three active substances of LDHJ granules and used in further cell experiments. The structural formulas of forsythoside-A (C_29_H_36_O_15_), emodin (C_15_H_10_O_5_), and chlorogenic acid (C_16_H_18_O_9_) were shown in **Figure 12B**. And the optimal experimental conditions for forsythoside-A and chlorogenic acid both were 40 µM for 24 h, while the selected optimal conditions for emodin were 20 µM for 24 h.

### Confirmation of the Protective Role of Three Active Substances of LDHJ Granules on the Cholestasis-Related Hepatocyte Apoptosis Through Regulating CaSR

#### The Supernatant Levels of Biomarkers Associated With Cholestasis and Liver Injuries

As illustrated in **Table 5**, primary hepatocytes treated with TCDC presented higher levels of the biomarkers (ALT, AST, γ-GT, ALP, TBIL, DBIL, and TBA) than the normal control group (*P* < 0.05, *P* < 0.01). However, the levels of these indices were attenuated by NPS-2390 (*P* < 0.05, *P* < 0.01). Similar results were obtained from cells treated with TCDC plus forsythoside-A, emodin and chlorogenic acid (*P* < 0.05, *P* < 0.01). Additionally, GdCl_3_ reversed the reductions in the levels of these indices induced by forsythoside-A, emodin or chlorogenic acid (*P* < 0.05, *P* < 0.01). Thus, forsythoside-A, emodin and chlorogenic acid ameliorated the TCDC-induced cholestasis and liver injuries by exerting effects similar to NPS-2390.

**Table 5:** Effects of forsythoside-A, emodin, and chlorogenic acid on the supernatant levels of biomarkers associated with cholestasis and liver injuries in primary hepatocytes.

Group	ALT	AST	γ-GT	ALP	TBIL	DBIL	TBA
control	0.57±0.03	2.37±0.11	5.16±0.32	0.54±0.01	4.28±0.11	0.69±0.03	24.44±0.56
TCDC	1.43±0.11^**^	5.52±0.17^**^	8.11±0.26^**^	0.74±0.01^*^	8.36±0.32^**^	1.61±0.13^**^	76.67±0.96^**^
TCDC+NPS-2930	0.97±0.06^#^	3.45±0.23^#^	5.90±0.29^#^	0.59±0.01^#^	5.19±0.33^##^	0.84±0.04^#^	36.67±0.96^##^
TCDC+forthoside A	0.64±0.09^##^	2.56±0.11^##^	5.16±0.16^#^	0.54±0.02^#^	4.28±0.40^##^	0.69±0.03^##^	25.56±2.00^##^
TCDC+forthoside A+GdCl3	1.10±0.07^@^	4.15±0.28^@@^	7.38±0.40^@^	0.66±0.01^@^	7.45±0.30^@@^	1.31±0.13^@@^	48.33±0.96^@^
TCDC+emodin	0.64±0.01^##^	2.43±0.06^##^	5.16±0.32^#^	0.54±0.01^#^	4.28±0.25^##^	0.71±0.08^##^	25.00±1.67^##^
TCDC+emodin+GdCl3	1.17±0.07^^^	4.48±0.11^^^^	7.37±0.37^^^	0.68±0.02^^^	7.00±0.36^^^^	1.30±0.07^^^^	51.11±2.65^^^
TCDC+chlorogenic acid	0.97±0.07^#^	3.31±0.11^#^	5.90±0.24^#^	0.61±0.01^#^	5.19±0.49^##^	0.89±0.08^#^	33.89±1.47^##^
TCDC+chlorogenic acid+GdCl3	1.50±0.11^&^	5.39±0.11^&&^	8.11±0.28^&^	0.74±0.01^&^	8.36±0.35^&&^	1.60±0.08^&&^	74.44±2.20^&&^

Data are presented as the means ± SD. n=3. LDHJ, Li-Dan-He-Ji; ALT, alanine aminotransferase; AST, aspartate aminotransferase; γ-GT, γ-glutamyl transpeptidase; ALP, alkaline phosphatase; TBIL, total bilirubin; DBIL, direct bilirubin; TBA, total bile acid. *P < 0.05 and ^**^P < 0.01 vs. the control group; ^#^P < 0.05 and ^##^P < 0.01 vs. the TCDC group; ^@^P < 0.05 and ^@@^P < 0.01 vs. the TCDC+ forsythoside-A group; ^P < 0.05 and ^^P < 0.01 vs. the TCDC+ emodin group; ^&^P < 0.05 and ^&&^P < 0.01 vs. the TCDC+ chlorogenic acid group.

#### Hepatocytic CaSR Expression and [Ca^2+^]_i_

**Figure 14:**
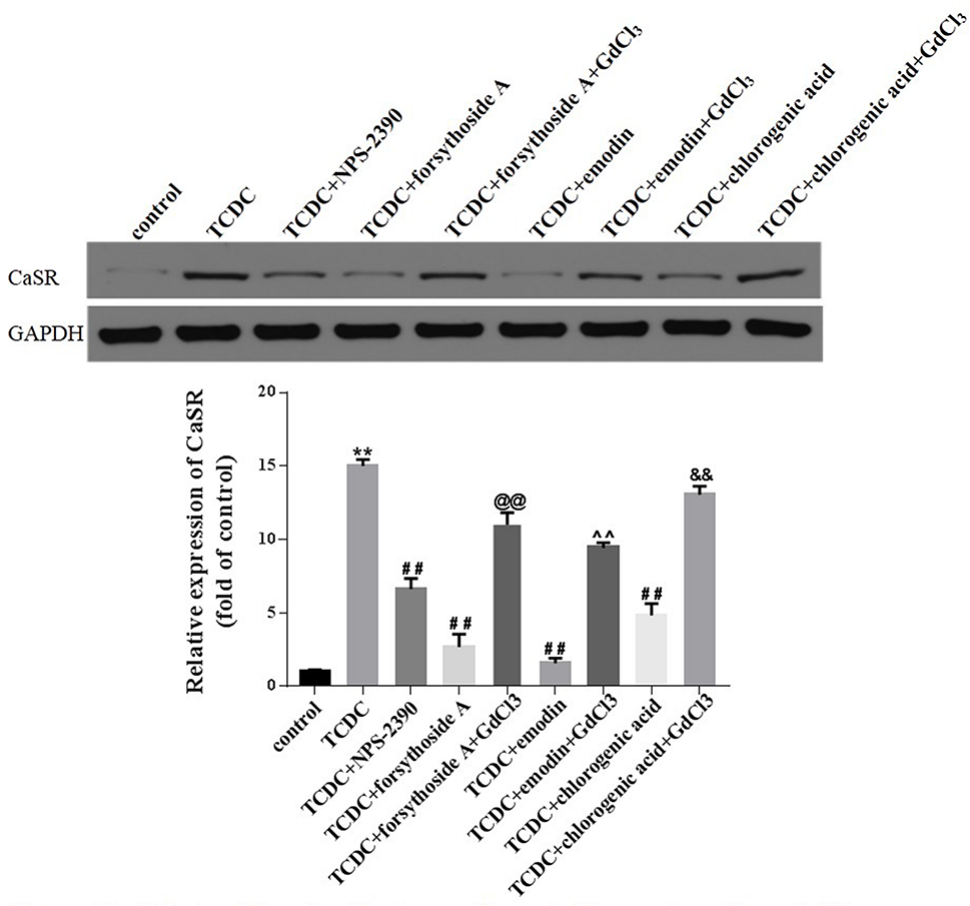
Effects of forsythoside-A, emodin and chlorogenic acid on CaSR expression *in vitro*. The levels of CaSR in primary hepatocytes were analyzed using the western blot analysis. GAPDH served as the internal control. **P < 0.01 vs the control group; ^##^ *P* < 0.01 vs the TCDC group; ^@@^ *P* < 0.01 vs the TCDC+forsythoside-A group; ^^^^ *P* < 0.01 vs the TCDC+emodin group; ^&&^ *P* < 0.01 vs the TCDC+chlorogenic acid group.

**Figure 15:**
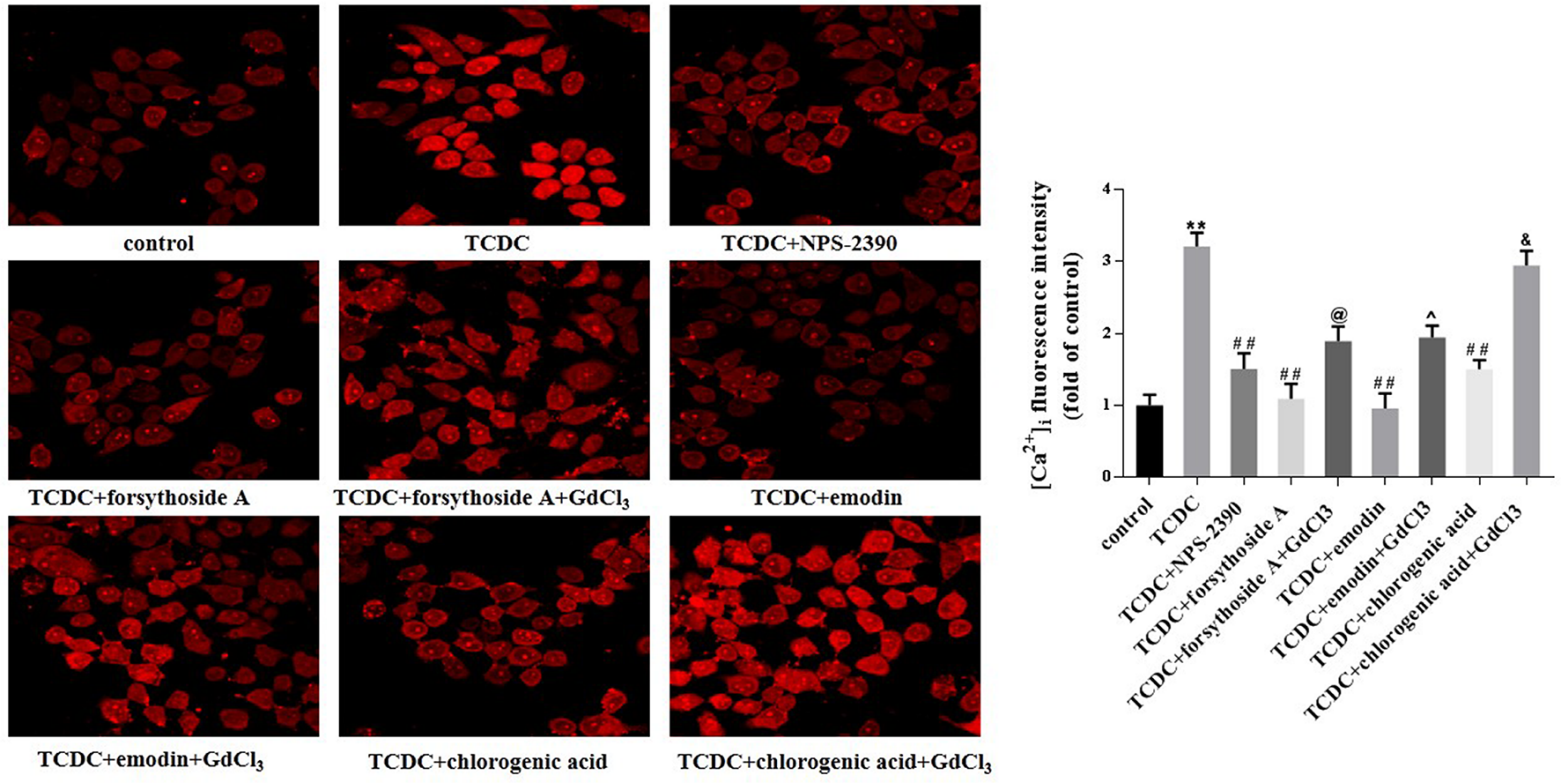
Effects of forsythoside-A, emodin and chlorogenic acid on [Ca^2+^]i in *vitro*. The fluorescence intensity of [Ca^2+^]_i_ in primary hepatocytes analyzed by LSCM. **P < 0.01 vs the control group; ^##^ *P* < 0.01 vs the TCDC group; ^@^ *P* < 0.05 vs the TCDC+forsythoside-A group; ^^^ *P* < 0.05 vs the TCDC+emodin group; ^&^ *P* < 0.05 vs the TCDC+chlorogenic acid group.

Western blot analyses showed increased levels of CaSR in the TCDC-treated group compared with the normal control group (**Figure 14**, *P* < 0.01). The administration of NPS-2390, forsythoside-A, emodin and chlorogenic acid decreased the levels of CaSR compared with the TCDC-treated group (**Figure 14**, *P* < 0.01). Groups that were subsequently treated with GdCl_3_ showed increased levels of CaSR (**Figure 14**, *P* < 0.01). LSCM was used to examine the [Ca^2+^]_i_ in the primary hepatocytes. TCDC markedly increased [Ca^2+^]_i_ compared to the normal control group (**Figure 15**, *P* < 0.01). However, these increases were reduced by further treatment with NPS-2390, forsythoside-A, emodin or chlorogenic acid (**Figure 15**, *P* < 0.01). Furthermore, the effects of forsythoside-A, emodin and chlorogenic acid on the [Ca^2+^]_i_ were attenuated when GdCl_3_ was added to the cells (**Figure 15**, *P* < 0.05). Taken together, forsythoside-A, emodin and chlorogenic acid decreased the levels of CaSR and [Ca^2+^]_i_ in the primary hepatocytes stimulated with TCDC.

#### Hepatocytic Levels of Apoptosis-Related Proteins Involved in the Mitochondrial Pathway

**Figure 16:**
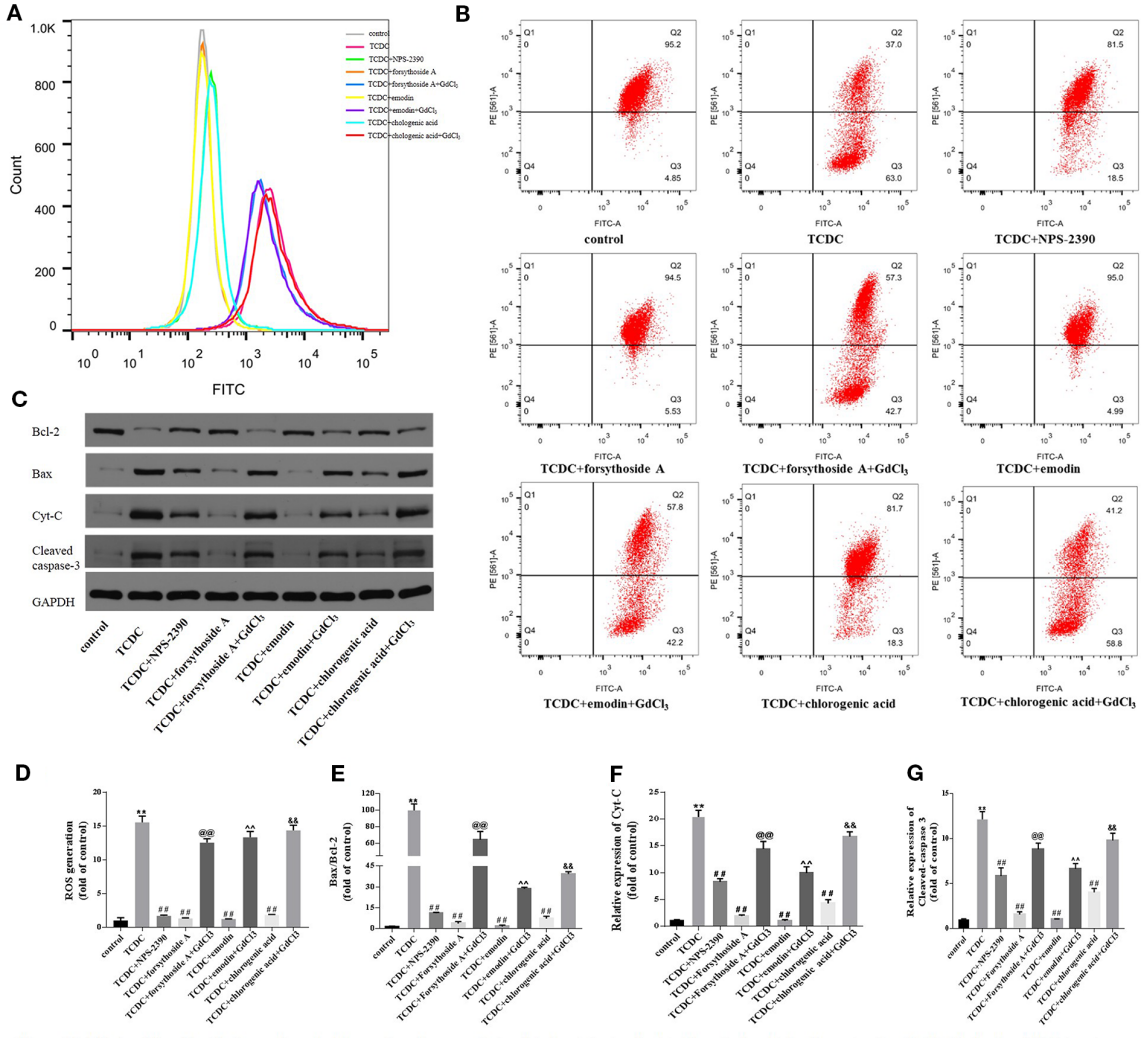
Effects of forsythoside-A, emodin, and chlorogenic acid on apoptosis-related proteins involved in the mitochondrial pathway *in vitro*. **(A, D)** The levels of ROS in primary hepatocytes. **(B)** Evaluation of MMP in primary hepatocytes. **(C, E–G)** The levels of Bax, Bcl-2, Cyt-C, and cleaved caspase-3 in primary hepatocytes were analyzed using western blot analysis. GAPDH served as the internal control. **P < 0.01 vs the control group; ^##^ *P* < 0.01 vs the TCDC group; ^@@^ *P* < 0.01 vs the TCDC + forsythoside-A group; ^^^^ *P* < 0.01 vs the TCDC + emodin group; ^&&^ *P* < 0.01 vs the TCDC + chlorogenic acid group.

Changes in the MMP, ROS generation, and expressions of mitochondrial apoptosis pathway-related proteins were detected to further confirm the involvement of the mitochondrial apoptosis pathway in hepatocyte apoptosis. The remarkable increases in ROS levels (**Figures 16A, D**, *P* < 0.01) and decreases in Δψm (**Figure 16B**) were observed in the TCDC-treated group compared with the normal control group. However, Δψm was increased and ROS levels were reduced in primary hepatocytes treated with the combination of TCDC and NPS-2390 compared with the TCDC-treated group (**Figures 16A, B, D**, *P* < 0.01). Similarly, forsythoside-A, emodin and chlorogenic acid also reversed the effects of TCDC on Δψm and ROS levels (**Figures 16A, B, D**, *P* < 0.01). As shown in **Figures 16 A, B, D**, further treatment with GdCl_3_ reduced Δψm and increased ROS levels in cells treated with forsythoside-A, emodin or chlorogenic acid (*P* < 0.01). Additionally, marked increases in the ratio of Bax/Bcl-2 and Cyt-C and cleaved caspase-3 levels were observed in the TCDC-treated group compared with the normal control group (**Figures 16C, E–G**, *P* < 0.01). However, the opposite results were obtained in hepatocytes treated with TCDC plus NPS-2390, forsythoside-A, emodin or chlorogenic acid (**Figures 16C, E–G**, *P* < 0.01). As shown in **Figures 16C, E–G**, after further treatment with GdCl_3_, changes in the Bax/Bcl-2 ratio and levels of Cyt-C and cleaved caspase-3 were reversed in primary hepatocytes treated with TCDC plus forsythoside-A, emodin or chlorogenic acid (*P* < 0.01).

#### Hepatocytic Levels of Apoptosis-Related Proteins Involved in the MAPK Pathway

**Figure 17:**
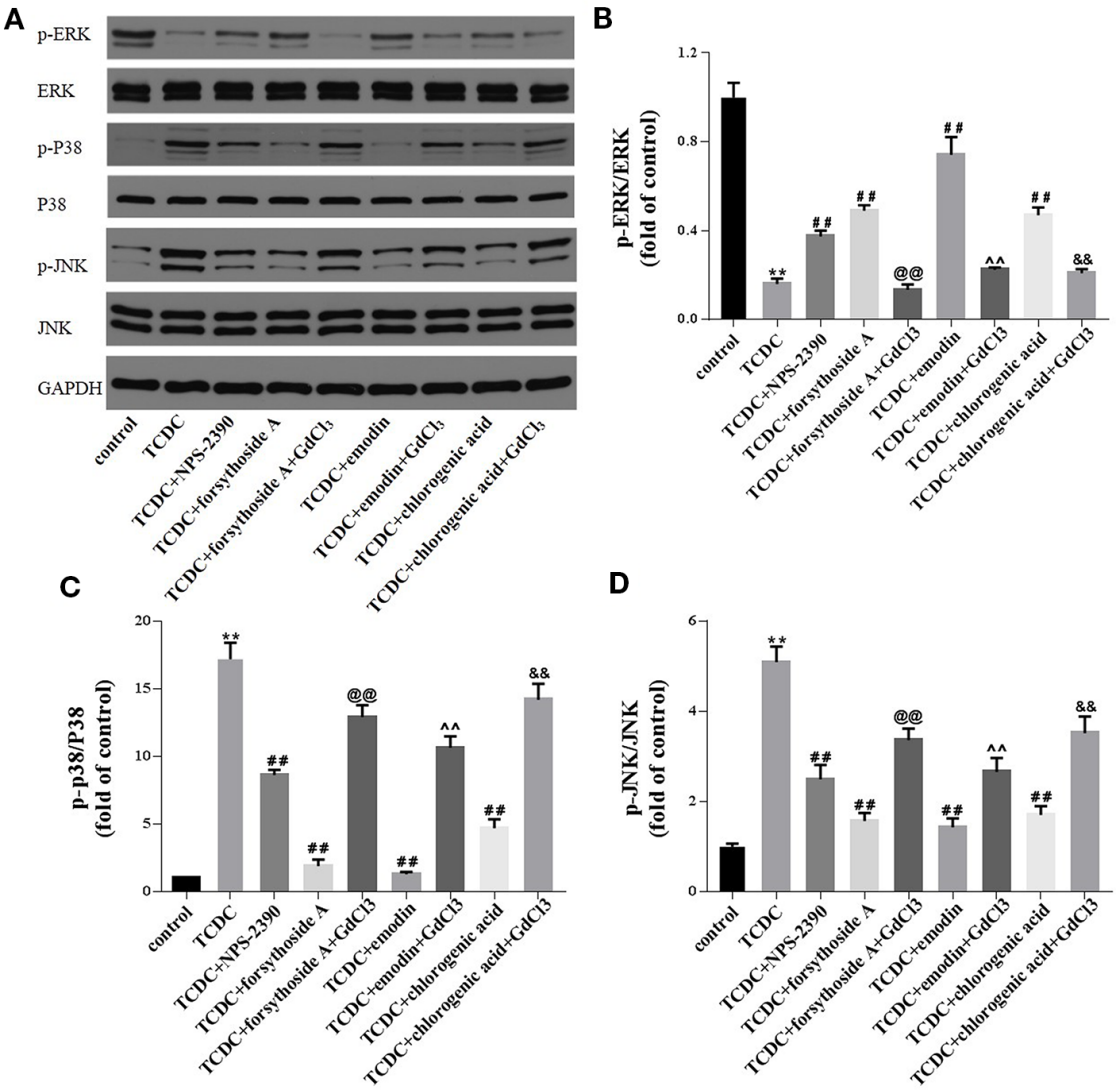
Effects of forsythoside-A, emodin and chlorogenic acid on apoptosis-related proteins involved in the MAPK pathway *in vitro*. **(A–D)** The levels of p-ERK, ERK, p-P38, P38, p-JNK, and JNK in primary hepatocytes were analyzed using western blot analysis. GAPDH served as the internal control. **P<0.01 vs the control group; ^##^ *P* < 0.01 vs the TCDC group; ^@@^ *P* < 0.01 vs the TCDC + forsythoside-A group; ^^^^ *P* < 0.01 vs the TCDC+emodin group; ^&&^ *P* < 0.01 vs the TCDC + chlorogenic acid group.

Western blot analyses were conducted to investigate whether forsythoside-A, emodin, and chlorogenic acid inhibits hepatocyte apoptosis through the MAPK pathway. The levels of p-JNK/JNK and p-P38/P38 were remarkedly increased in the TCDC-treated group compared with the normal control group (**Figures 17A, C, D**, *P* < 0.01), and these changes were reversed by NPS-2390, forsythoside-A, emodin and chlorogenic acid (**Figures 17A, C, D**, *P* < 0.01). Conversely, the levels of the p-ERK/ERK proteins were significantly decreased in the TCDC-treated group compared with the normal control group (**Figure 17B**, *P* < 0.01). Cotreatment with NPS-2390, forsythoside-A, emodin or chlorogenic acid significantly increased p-ERK levels in the TCDC-induced intrahepatic cholestasis model (**Figure 17B**, *P* < 0.01). Further treatment with GdCl_3_ produced the opposite results (**Figures 17A–D**, *P* < 0.01).

## Discussion

Plant-based medicines are widely used to treat jaundice in the local clinics of China. In Chinese medicine, ICH is referred to as “foetal jaundice disease” (China Association of Chinese Medicine, 2012). LDHJ was developed by the Department of Integrated Traditional Chinese and Western Medicine, Wuhan Children’s Hospital, Tongji Medical College, Huazhong University of Science and Technology based on 30 years of experience in treating “foetal jaundice disease”. LDHJ is a compound preparation consisting of *Forsythia suspensa* (Thunb.) Vahl (20 g), *Artemisia capillaries* Thunb. (30 g), *Rheum palmatum* L. (processing with rice wine) (5 g), *Schisandra sphenanthera* Rehd. et Wils. (10 g), *Paeonia lactiflora* Pall. (30 g), *Atractylodes macrocephala* Koidz. (10 g), *Citrus aurantium* L. (10g), *Glycyrrhiza uralensis* Fisch (5 g), *Polygonum multiforum* Thunb. (5 g), *Cinnamomum cassia* Presl (5 g), and *Manis pentadactyia* Linnaeus (3 g). Previously, our research group administered LDHJ to infants with cytomegalovirus infection induced-ICH and neonatal intra-hepatic cholestasis caused by citrin defects. After completing a number of clinical trials, LDHJ has shown a satisfactory clinical efficacy in relieving cholestasis (Yan et al., 2012; Zhang et al., 2017). In addition, we also conducted a study examining the dose-dependent effects of different dosages of LDHJ on intrahepatic cholestasis in young rats and found that multiple-dose groups of *Forsythia suspensa* (Thunb.) Vahl, *Rheum palmatum* L. (processing with rice wine), and *Artemisia capillaries* Thunb. were superior to the group treated with a constant dose of LDHJ, indicating that the predominate drugs of LDHJ *Forsythia suspensa* (Thunb.) Vahl, *Rheum palmatum* L. (processing with rice wine), and *Artemisia capillaries* Thunb. played a leading role in the whole prescription (Zhang et al., 2019). As mentioned above, severe and persistent cholestasis leads to liver fibrosis, cirrhosis, and liver failure; thus, patients ultimately require a liver transplant (Lu, 2015). Therefore, in this study, we first preliminarily investigated the possible mechanism by which LDHJ improved cholestasis and protected hepatocytes *in vivo* and then verified the aforementioned mechanism of the three active substances: forsythoside-A from *Forsythia suspensa* (Thunb.) Vahl, emodin from *Rheum palmatum* L. (processing with rice wine), chlorogenic acid from *Artemisia capillaries* Thunb. *in vitro*.

*In vivo* study, we used ANIT to establish the rat model of hepatotoxicity (Ding et al., 2016; Xiong et al., 2019) and evaluate the effects of LDHJ granules on intrahepatic cholestasis (Kossor et al., 1993). Consistent with the previously reported findings (Wang et al., 2014), the increased levels of biomarkers (TBA, TBIL, DBIL, AST, ALT, ALP, and γ-GT) and abnormal histopathological changes were observed in the liver of young rats with ANIT-induced intrahepatic cholestasis. Additionally, LDHJ granules reversed these changes. Taken together, these findings reveal the protective effects of LDHJ granules on ANIT-induced intrahepatic cholestasis. Subsequently, we evaluated the hepatocyte apoptosis in rats by TUNEL staining. The green coloration proved that hepatocyte apoptosis exists in ANIT-induced intrahepatic cholestasis model rats. However, the apoptotic rate became lower after LDHJ treatment indicating that LDHJ is effective on ameliorating liver apoptosis in intrahepatic cholestasis.

Hepatic mitochondria have been identified as a major target of hepatotoxicity induced bile acids in subjects with cholestasis (Heidari et al., 2018). As reported in a previous study, the mitochondrial pathway plays a crucial role in cell apoptosis (Gibson and Davids, 2015). Based on accumulating evidence, activated CaSR is involved in the release of Ca^2+^ from the mitochondria and subsequently participates in the cell apoptotic mechanism (Wendt-Gallitelli and Isenberg, 1992). In the present study, elevated levels of the CaSR protein were observed in the liver of ANIT-induced rats, while LDHJ granules inhibited the activation of CaSR. Members of the Bcl-2 family, including pro-apoptotic proteins (such as Bax) and anti-apoptotic proteins (such as Bcl-2), are the most crucial regulators of cell apoptosis by acting directly on the mitochondria (Martinou and Youle, 2011; Huang et al., 2018). Briefly, an increase in the Bax/Bcl-2 ratio increases the permeability of the mitochondrial membrane, leading to the release of Cyt-C from the mitochondria to the cytoplasm. The released Cyt-C elicits the sequential activation of cytosolic caspase-9 and caspase-3, followed by the activation of the caspase-independent mitochondrial pathway. Yao et al. reported a role for the mitochondrial pathway in ANIT-induced hepatocellular apoptosis (Yao et al., 2017). In the present study, LDHJ granules noticeably decreased the Bax/Bcl-2 ratio and the levels of Cyt-C and cleaved caspase-3 in the liver of ANIT-induced rats. Collectively, LDHJ granules functions as an antagonist of CaSR to restrain cell apoptosis by inhibiting the mitochondrial pathway in intrahepatic cholestasis models.

CaSR has also been reported to be associated with the MAPK pathway (Guo et al., 2012). ERK, JNK, and P38 kinase are three types of MAPK proteins involved in signalling pathways (Cao et al., 2013). Previous studies have reported a crucial role for MAPK in cell apoptosis (Zheng et al., 2014; Yuan et al., 2017). In particular, the involvement of P38, ERK, and JNK have been verified in models of cholestasis induced by oxidative stress (Toledo et al., 2017). In the present study, changes in the levels of p-P38/P38, p-ERK/ERK, and p-JNK/JNK were detected in the liver of ANIT-induced rats. Nevertheless, LDHJ granules reversed these changes, indicating that the inhibitory effects of LDHJ granules on cell apoptosis are mediated by the MAPK pathway.

In the current study, we further confirm the protective effects of LDHJ on cholestasis-related hepatocyte apoptosis through downregulating of CaSR and participating in the mitochondrial pathway and MAPK pathway. Firstly, we determined the positive changes of CaSR expression and apoptosis in four primary hepatocyte models as follows: upregulated hepatocyte CaSR expression; TCDC-induced hepatocyte apoptosis in cholestasis; upregulated hepatocyte CaSR expression in TCDC-induced hepatocyte apoptosis in cholestasis and downregulated hepatocyte CaSR expression in TCDC-induced hepatocyte apoptosis in cholestasis. Secondly, three active substances of LDHJ (forsythoside-A, emodin and chlorogenic acid) were screened and used in cell experiments.

*In vitro*, both liver injury-related (AST and ALT) and cholestasis-related (γ-GT, ALP, TBIL, DBIL, and TBA) biomarkers were increased in the primary hepatocytes treated with TCDC. Meanwhile, the expression of CaSR elevated and hepatocyte apoptosis also trigged. Moreover, apoptosis was happened in CaSR overexpression primary hepatocyte model, was promoted in CaSR upregulation combined with TCDC-stimulated hepatocyte model, and was abolished in the CaSR downregulation combined with TCDC-stimulated hepatocyte model. Overall, these results testify the CaSR may be crucial in leading to cholestasis-related hepatocyte apoptosis. The protective effects of forsythoside-A and chlorogenic acid on cell apoptosis have been reported (Ali et al., 2017; Yan et al., 2017). Our research group has found the rescue activity of emodin on the intrahepatic cholestasis (Ding et al., 2016; Xiong et al., 2019). So forsythoside-A, emodin, chlorogenic acid may be promising in representing the LDHJ for further cell experiments. In our vitro study, we compared with eight main substances of LDHJ granules in regulating the expression of CaSR and hepatocyte apoptosis in TCDC-stimulated primary hepatocytes. As what we expected, the aforementioned three active substances (forsythoside-A, emodin, chlorogenic acid) were screen out. All of them could ameliorate the intrahepatic cholestasis, as verified by the decreased levels of liver injury and cholestasis-related biomarkers, downregulate CaSR expression and inhibit hepatocyte apoptosis.

As mentioned in *in vivo* study, both the mitochondrial pathway and MAPK pathway are associated with cholestasis-related hepatocyte apoptosis caused by CaSR. The trend of changes of re-estimated items related to the aforementioned pathways (mitochondrial pathway: the Bax/Bcl-2 ratio, the Cyt-C and cleaved caspase-3 levels; MAPK pathway: p-P38/P38, p-ERK/ERK, and p-JNK/JNK ratio) in all three active substances treatments *in vitro* were consistent with that of LDHJ treatment *in vivo*.

Moreover, *in vitro* study, we added extra items to estimate downstream of CaSR and it mediated mitochondrial pathway. The accumulated bile acids might alter the MMP and ROS generation to subsequently exert some adverse effects on the biological function of mitochondria, leading to increased cell apoptosis (de-Andrade et al., 2015). Notably, the loss of MMP was also observed in the TCDC-induced cholestasis-related hepatocyte apoptosis. However, the opposite results were obtained from in the cell model of cholestasis-related hepatocyte apoptosis treated with the three active substances. The mitochondria are the main cellular source of ROS. Accumulated bile acids have been reported to cause hepatocyte apoptosis in humans with cholestasis through ROS-mediated oxidative stress (Toledo et al., 2017). Meanwhile, calcium overload caused by CaSR is also implicated in cell apoptosis (Guo et al., 2012; Qi et al., 2013). Furthermore, the important roles of [Ca^2+^]_i_ and ROS in cell apoptosis mediated by the mitochondrial pathway have been documented. For instance, crocin inhibits osteoblast apoptosis by stimulating the ROS/Ca^2+^−mediated mitochondrial pathway (Nie et al., 2019). In the present study, [Ca^2+^]_i_ and ROS levels were initially detected to explore the more specific role of CaSR in the cholestasis-related hepatocyte apoptosis. The three active substances reduced the increases in [Ca^2+^]_i_ and ROS levels in the cell model of cholestasis-related hepatocyte apoptosis. Thus, the three active substances of LDHJ granules inhibited CaSR expression involved in calcium overload and ROS generation *in vitro*.

In addition, we also added GdCl_3_ (agonist of CaSR) to pre-stimulate model hepatocytes before the three active substances intervention respectively. Cotreatment with GdCl_3_ reversed the positive effects of forsythoside-A, emodin and chlorogenic acid on attenuating the hepatocyte cholestasis and injury, relieving the hepatocyte apoptosis, inhibiting the expression of hepatocyte CaSR and regulating the molecules involved in CaSR-mediated mitochondrial and MAPK pathway.

In summary, LDHJ improved cholestasis, downregulated CaSR expression and inhibited hepatocyte apoptosis in the young animal model with intrahepatic cholestasis through regulating the mitochondrial pathway and MAPK pathway, and further cell experiments show that one of the three active substances (forsythoside-A, emodin and chlorogenic acid) may be the inhibitors of CaSR.

At the end, we are aware of the limitation of the present study. First of all, LDHJ consist of 11 Chinese herbs. It is difficult to fully understand the mechanisms of LDHJ *via* one research due to its complicated ingredients. TCM treatments generally have multiple targets and act on multiple pathways. Nowadays the nuclear receptors such as farnesoid X receptor (FXR) or pregnane X receptor (PXR), have been recognized as potential therapeutic targets in cholestasis (Gonzalez-Sanchez et al., 2015; Beuers et al., 2015). Our group have carried out some studies on emodin alleviating intrahepatic cholestasis by regulation of liver FXR pathway (Ding et al., 2016; Xiong et al., 2018; Xiong et al., 2019). The exploration of LDHJ on FXR target and relevant transporters and synthesic enzymes of bile acid is in progress and the results will be reported later. Second of all, forsythoside-A, emodin and chlorogenic acid are structural different compounds, but they have shown similar effects against CaSR in this study. Hence, which one of those three active substances directly inhibit CaSR to be further validate in upregulated and downregulated CsSR expression hepatocytes models. Third and last, the comparison between combination treatment of forsythoside-A, emodin, and chlorogenic acid according to the actual content ratio in LDHJ and single treatment of those three active substances is not considered in current cell experiments.

## Data Availability Statement

The raw data supporting the conclusions of this article will be made available by the authors, without undue reservation, to any qualified researcher.

## Ethics Statement

The animal study was reviewed and approved by The Institutional Animal Care and Use Committee at Tongji Medical College, Huazhong University of Science and Technology.

## Author Contributions

HQ, X-lX, L-sZ, and S-qY designed the experiments. Ling-lZ, Z-xJ, Lin-lZ, and Y-tW performed the experiments. Y-yQ acquired the data. Y-jW and C-pX analyzed the data. L-sZ and S-qY wrote and revised the manuscript.

## Funding

This study was supported by grants from the National Natural Science Foundation of China (NO. 81574024), Clinical medical research project in 2017, Health and Family Planning Commission of Wuhan Municipality (NO. WZ17A07), and General Scientific Research Project of Integrated Traditional Chinese and Western Medicine from 2016 to 2017, Health and Family Planning Commission of Hubei Province (NO. 2017HZ-Y35).

## Conflict of Interest

The authors declare that the research was conducted in the absence of any commercial or financial relationships that could be construed as a potential conflict of interest.
